# Cell Cycle Control by the Master Regulator CtrA in *Sinorhizobium meliloti*

**DOI:** 10.1371/journal.pgen.1005232

**Published:** 2015-05-15

**Authors:** Francesco Pini, Nicole J. De Nisco, Lorenzo Ferri, Jon Penterman, Antonella Fioravanti, Matteo Brilli, Alessio Mengoni, Marco Bazzicalupo, Patrick H. Viollier, Graham C. Walker, Emanuele G. Biondi

**Affiliations:** 1 Unité de Glycobiologie Structurale et Fonctionnelle, UMR8576 CNRS—Université de Lille, Villeneuve d'Ascq, France; 2 Department of Biology, Massachusetts Institute of Technology, Cambridge, Massachusetts, United States of America; 3 Meyer Children Hospital, University of Florence, Firenze, Italy; 4 Fondazione Edmund Mach/CRI, Functional genomics, San Michele all'Adige, Italy; 5 Dept. of Biology, University of Florence, Firenze, Italy; 6 Dept. Microbiology & Molecular Medicine, University of Geneva, Genève, Switzerland; Universidad de Sevilla, SPAIN

## Abstract

In all domains of life, proper regulation of the cell cycle is critical to coordinate genome replication, segregation and cell division. In some groups of bacteria, *e*.*g*. *Alphaproteobacteria*, tight regulation of the cell cycle is also necessary for the morphological and functional differentiation of cells. *Sinorhizobium meliloti* is an alphaproteobacterium that forms an economically and ecologically important nitrogen-fixing symbiosis with specific legume hosts. During this symbiosis *S*. *meliloti* undergoes an elaborate cellular differentiation within host root cells. The differentiation of *S*. *meliloti* results in massive amplification of the genome, cell branching and/or elongation, and loss of reproductive capacity. In *Caulobacter crescentus*, cellular differentiation is tightly linked to the cell cycle via the activity of the master regulator CtrA, and recent research in *S*. *meliloti* suggests that CtrA might also be key to cellular differentiation during symbiosis. However, the regulatory circuit driving cell cycle progression in *S*. *meliloti* is not well characterized in both the free-living and symbiotic state. Here, we investigated the regulation and function of CtrA in *S*. *meliloti*. We demonstrated that depletion of CtrA cause cell elongation, branching and genome amplification, similar to that observed in nitrogen-fixing bacteroids. We also showed that the cell cycle regulated proteolytic degradation of CtrA is essential in *S*. *meliloti*, suggesting a possible mechanism of CtrA depletion in differentiated bacteroids. Using a combination of ChIP-Seq and gene expression microarray analysis we found that although *S*. *meliloti* CtrA regulates similar processes as *C*. *crescentus* CtrA, it does so through different target genes. For example, our data suggest that CtrA does not control the expression of the Fts complex to control the timing of cell division during the cell cycle, but instead it negatively regulates the septum-inhibiting Min system. Our findings provide valuable insight into how highly conserved genetic networks can evolve, possibly to fit the diverse lifestyles of different bacteria.

## Author Summary

In order to propagate, all living cells must ensure that their genetic material is faithfully copied and properly partitioned into the daughter cells before division. These coordinated processes of DNA replication and cell division are termed the “cell cycle” and are controlled by a complex network of regulatory proteins in all organisms. In the class *Alphaproteobacteria*, the regulation of the cell cycle is closely linked to cellular differentiation processes that are vital for survival in the environment. In these bacteria, the cell cycle regulator CtrA is thought to serve as the primary link between the coordination of the cell cycle and cellular differentiation. The alphaproteobacterium, *Sinorhizobium meliloti*, an important model symbiont of alfalfa plants, undergoes a striking cellular differentiation that is vital to the formation of an efficient symbiosis dedicated to the conversion of atmospheric nitrogen to biologically available organic nitrogen. However, the link between cellular differentiation and cell cycle control in *S*. *meliloti* has not been made. In this study, we showed that *S*. *meliloti* cells without CtrA are similar to the symbiotic form. By the identification of the genes whose expression is directly and indirectly controlled by CtrA, we found that CtrA regulates vital cell cycle processes, including DNA replication and cell division, but through different genetic pathways than in other alphaproteobacteria. We importantly show that the levels of CtrA protein are governed by an essential cell cycle regulated proteolysis, which may also be an important mode of CtrA down-regulation during symbiosis to drive cellular differentiation.

## Introduction

The alphaproteobacterium *Sinorhizobium meliloti* can thrive in the soil as a free-living organism or as a nitrogen-fixing symbiotic partner with compatible legume hosts [1]. The *S*. *meliloti*-legume symbiosis involves multiple developmental stages, during which the bacteria coordinate their cell proliferation with the development of the host plant cells [2,3]. A key step in this symbiosis is the striking differentiation of *S*. *meliloti* cells into enlarged, polyploid (16–32 copies of the genome) nitrogen-fixing bacteroids within the specialized host cells that comprise the developing nodule [4]. Differentiation of bacteroids in *S*. *meliloti*-legume symbiosis is driven in part by nodule specific cysteine-rich peptides (NCRs) that are produced by the host legume [5,6]. These peptides, such as NCR247, can provoke in free-living cells many of the changes associated with bacteroid differentiation including the increase in cell size and endoreduplication of the genome [7]. The uncoupling of DNA replication from cell division in *S*. *meliloti* during symbiosis stands in stark contrast to the cell cycle of free-living *S*. *meliloti*, where DNA replication is tightly coupled to cell division [8].

The involvement of the cell cycle regulatory network in cellular differentiation programs, such as cyst formation in *Rhodospirillum centenum* and the asymmetric division of *Caulobacter crescentus*, is a common theme in *Alphaproteobacteria* [9,10]. In *C*. *crescentus* and presumably in other alphaproteobacteria, cellular differentiation is largely governed by the response regulator CtrA [10–14]. *C*. *crescentus* divides asymmetrically to produce two morphologically different cells, a motile swarmer cell and a sessile stalked cell [15]. The two cell types are also distinct in their replicative capacities. The stalked cell, which lacks active CtrA, can immediately initiate DNA replication and re-enter the cell cycle, while in the swarmer cell, the origin of replication is bound and inhibited by phosphorylated CtrA resulting in a G1 arrest [16,17]. As a transcription factor, phosphorylated CtrA binds *ca*. 200 promoter regions and controls the transcription of about 95 genes over the course of the cell cycle, thereby modulating diverse processes including polar morphogenesis and cell division [18]. The expression, activity and stability of *C*. *crescentus* CtrA are highly regulated during the cell cycle through transcriptional regulation, phosphorylation and regulated proteolysis [19–27].

An essential, functional homolog of *C*. *crescentus* CtrA is present in *S*. *meliloti* and has been implicated in the symbiotic cellular differentiation program [28]. The genetic circuit controlling CtrA in *S*. *meliloti* at the transcriptional and posttranslational levels has been predicted using bioinformatics and all the regulatory factors identified in *C*. *crescentus* are conserved on the sequence level in *S*. *meliloti* [11]. Genetic experiments on a few of these putative regulators of CtrA have revealed a striking link between symbiosis and cell cycle regulation [29–33]. In addition, gene expression profiling of *S*. *meliloti* at different stages of the symbiosis indicated that expression of *ctrA* is strongly down-regulated in bacteroids once differentiation begins [34], and Western blot analysis of purified bacteroids revealed that CtrA protein levels are very low in mature bacteroids [33]. More specifically, down regulation of CtrA during symbiosis may be caused by exposure to NCR peptides, as *in vitro* treatment of *S*. *meliloti* with a sub-lethal dose of the NCR peptide, NCR247, significantly attenuates *ctrA* expression [35]. Collectively, these observations suggest that NCR peptides and perhaps other plant factors modulate the cell cycle in part by affecting the level of CtrA activity. It is thus crucial to gain a deeper understanding of the factors governing the *S*. *meliloti* cell cycle, especially of the cell processes governed by CtrA and the regulatory mechanisms controlling CtrA activity.

In this study, we sought to understand the mechanisms regulating cell cycle regulation in *S*. *meliloti* by analyzing the effects of CtrA depletion in *S*. *meliloti* free-living cells. We aimed to define the direct and indirect transcriptional regulons of *S*. *meliloti* CtrA and probing regulatory mechanisms, such as regulated proteolysis that possibly govern CtrA levels during the *S*. *meliloti* cell cycle. As global analysis of the CtrA transcriptional network has not been performed in detail in an alphaproteobacterium other than *C*. *crescentus*, this work provides the first insight into how this highly conserved genetic network can evolve to fit the distinct lifestyles of this diverse group of bacteria. Furthermore, the model of CtrA cell cycle regulation in *S*. *meliloti* developed in this work will be pivotal in the future elucidation of how the bacterial cell cycle is modulated by plant factors during the symbiosis.

## Results

### Depletion of CtrA in *S*. *meliloti* causes “bacteroid-like” cell cycle changes

The current working model of cell cycle regulation in *Alphaproteobacteria* is largely based on the regulatory interactions identified in *C*. *crescentus*, thanks to their level of conservation in other species [11]. Cell cycle regulation in *C*. *crescentus*, especially the governance of replicative and morphological asymmetry, is centered on the master regulator CtrA, which can inhibit DNA replication initiation by directly binding the origin of replication and also acts as a transcriptional regulator regulating hundreds of genes [18]. Although CtrA is highly conserved in *S*. *meliloti*, the activity of CtrA as a transcription factor and the role of CtrA in regulating cell cycle functions have not been investigated. Therefore, in order to more clearly understand the role of CtrA in cellular differentiation during symbiosis, we focused our investigation on understanding the role of CtrA as a master regulator of the cell cycle in *S*. *meliloti*.

**Fig 1:**
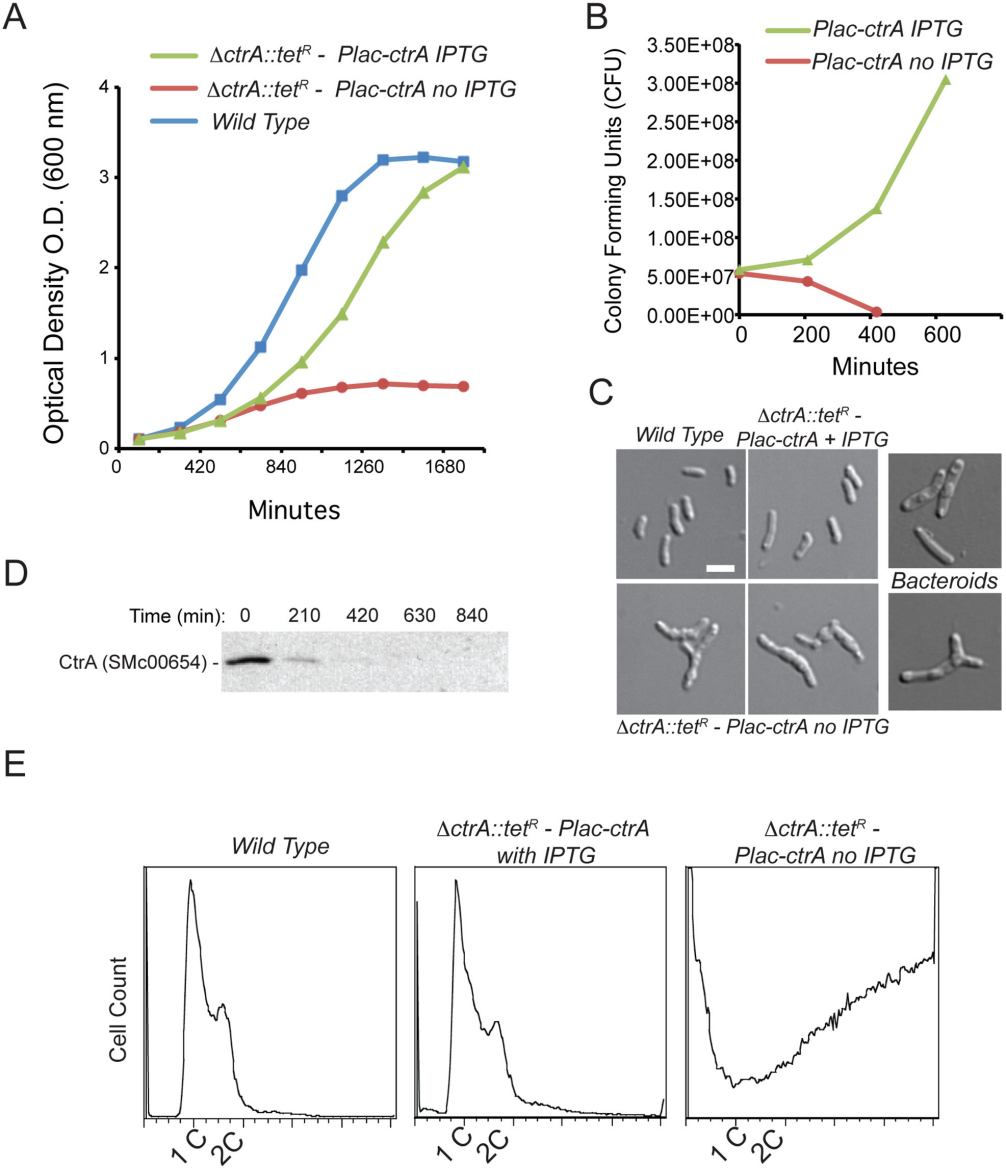
CtrA plays an essential role in *S*. *meliloti*. A. Optical density (OD_600_) of wild type *S*. *meliloti* and the CtrA depletion strain grown with and without IPTG, error bars represent standard errors. Mid-log phase cells depleted of CtrA show a stable OD level suggesting an impairment of normal growth. B. CFU of the experiments in (A) showing that cells without *ctrA* expression lost viability. C. Morphology of *S*. *meliloti* after 7 hours of CtrA depletion compared with wild type and bacteroid *S*. *meliloti*; cells appear elongated and enlarged (bar corresponds to 2 μm). D. Immunoblot analysis using anti-CtrA antibodies over a time course of CtrA depletion. E. FACS analysis of *S*. *meliloti* CtrA depletion strain after 8 hours +IPTG (control) and—IPTG (CtrA depleted) showing increased DNA content of up to 20 copies per cell in cells depleted of CtrA.

Previous work has demonstrated that *ctrA* (SMc00654) is an essential gene in *S*. *meliloti* [28]. To study the effects of loss of CtrA function in *S*. *meliloti* we constructed a conditional CtrA depletion strain utilizing the pSRK expression system based on IPTG induction [36]. We transduced a marked deletion of *ctrA* (Δ*ctrA*) to cells harboring an IPTG-inducible copy of *ctrA* on the pSRK-Km vector to create the strain *ΔctrA-P*_*lac*_ *ctrA* (see methods) (S1 Table). In the presence of 1mM IPTG, *ΔctrA-P*_*lac*_ *ctrA* cells were viable, exhibited normal morphology (Fig 1A–1C), and produced CtrA (S1 Fig). However, upon removal of IPTG, *ΔctrA*-P_*lac*_ *ctrA* cells ceased growth, lost viability and developed aberrant morphologies (Fig 1A–1C). These phenotypic changes coincided with the rapid decrease of CtrA protein over time to undetectable levels (Fig 1D). Cells depleted of CtrA were elongated, swollen and a fraction of cells became Y-shaped (Fig 1C). The phenotypic effects caused by CtrA depletion are reminiscent of the morphology of differentiated bacteroids in which CtrA is also absent (Fig 1C) [33]. Interestingly, despite these aberrant morphologies, cells depleted of CtrA maintained their membrane integrity, as indicated by the lack of propidium iodide incorporation (S2 Fig).

To quantify the observed genome amplification we measured DNA content using flow cytometry and found a striking increase in DNA content in CtrA-depleted cells compared to wild type cells and *ΔctrA*-P_*lac*_ *ctrA* cells grown in the presence of IPTG (Fig 1E). CtrA-depleted cells contained ca. 20 times the amount of DNA per cell as *ΔctrA-P*_*lac*_ *ctrA* cells supplemented with IPTG as well as wild type, log phase *S*. *meliloti* cells. In CtrA-depleted cells the 1N and 2N peaks were lost indicating a complete de-coupling of DNA replication initiation and cell division, indicating that CtrA serves as an essential link between these two processes. Unlike the *C*. *crescentus* paradigm, the *S*. *meliloti* chromosomal origin of replication does not contain CtrA-binding motifs [11] and is not bound by CtrA protein (see S4 Table), so if CtrA governs DNA replication initiation it likely does so through an indirect, unknown mechanism. Finally, we used qPCR to determine the ratio of the three replicons that comprise the *S*. *meliloti* genome and found that the ratio between the replicons did not significantly change after 4 hours of CtrA depletion (S3 Fig). These observations indicate that CtrA function is required to equally repress the replication of all *S*. *meliloti* three replicons.

Collectively these data support the hypothesis down-regulation of CtrA activity during symbiosis could contribute to the elongation and genome amplification observed in differentiating bacteroids [28,33,35].

### CtrA regulates the transcription of at least 126 genes in *S*. *meliloti*

**Fig 2:**
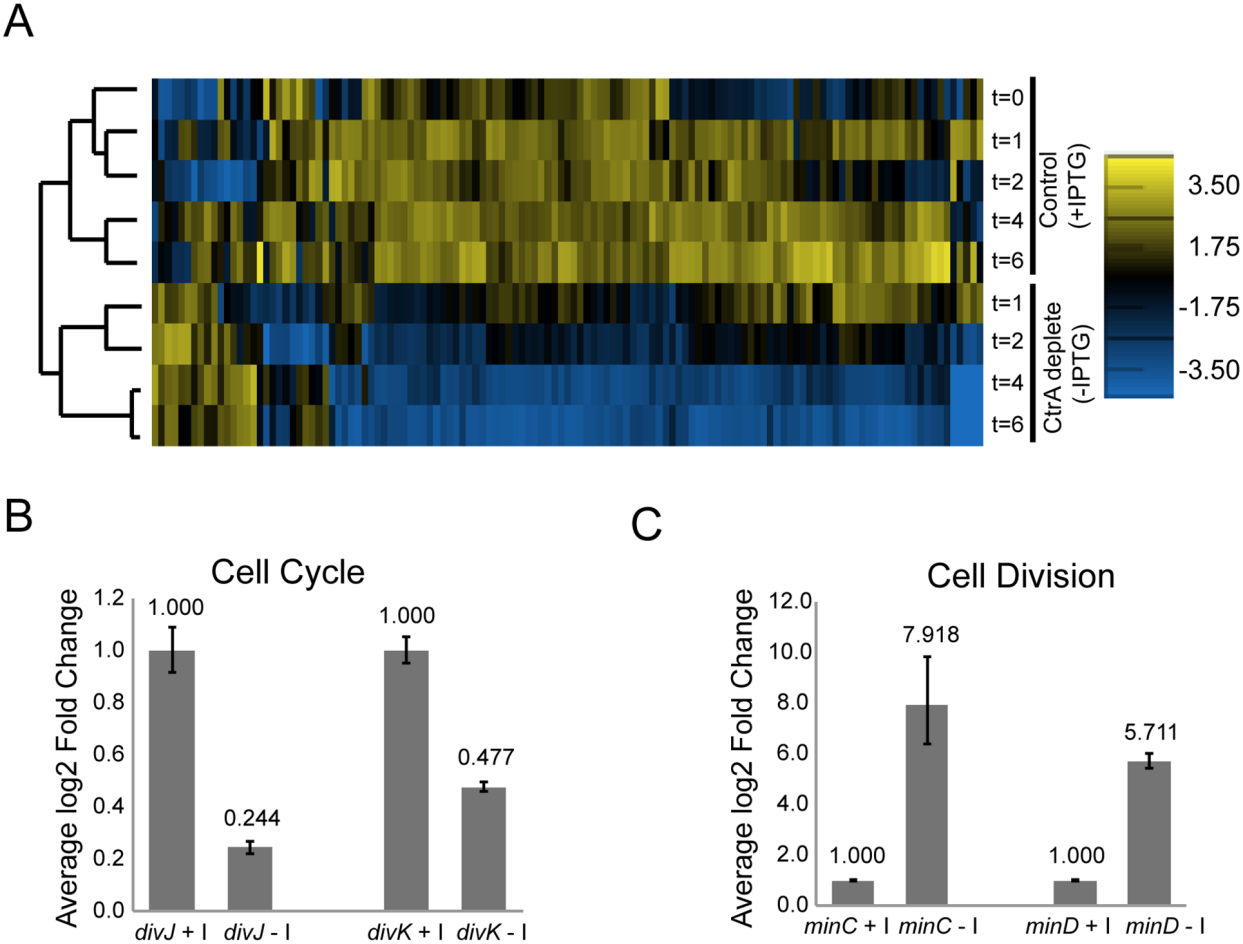
CtrA regulates the expression of at least 126 *S*. *meliloti* genes. A. Hierarchical clustered expression profiles for 126 genes in cells expressing *ctrA* (control; +IPTG) and in cells depleted of *ctrA* (-IPTG) at several time points (t = 0, 1, 2, 4 and 6 hours) following the initiation of the—IPTG or +IPTG treatment. Normalized log2 expression levels are shown for each gene. The scale for expression level is located on the right. B. Fold change in *divJ* and *divK* expression in cells after depletion of CtrA (-I, IPTG) for two hours relative to control cells expressing CtrA (+I). Expression of *divJ* and *divK* in each sample was normalized to the expression of the control gene *smc00128*. Shown are data from a representative biological replicate. Error bars indicate standard error. C. Fold change in *minC* and *minD* expression in cells after depletion of CtrA (-I, IPTG) for four hours relative to control cells expressing CtrA (+I). Data normalization was performed as in B. Shown are data from a representative biological replicate. Error bars indicate standard error.

To determine how the activity of CtrA as a transcription factor affects important cell cycle functions, we used microarray analysis to measure gene expression changes upon CtrA depletion in *S*. *meliloti* (S2 Table). For this experiment, exponential phase cultures of *ΔctrA-P*_*lac*_-*ctrA S*. *meliloti* grown in the presence of 1mM IPTG were washed and split into control (+IPTG) and *ctrA* depletion (–IPTG) sub-cultures. RNA was isolated from these cultures at different time points post-split and used for microarray-based gene expression analysis. We found that the expression of 126 genes changed significantly during CtrA depletion (Fig 2A, S3 Table). We validated the results by testing the expression of several key differentially expressed genes using qPCR and reporter *lacZ* fusions and found similar expression patterns as was detected by the microarray experiments (Fig 2B and S4A Fig). Hierarchical clustering of the expression data from each time point showed strong correlation between gene expression in control vs. CtrA depleted samples and a strong temporal effect of CtrA depletion on gene expression (Fig 2A).

Differentially expressed genes were found on each of the three *S*. *meliloti* replicons, with the majority (80%) located on the chromosome (S2 Table). Most of the genes (~86%) affected by depletion of CtrA were down-regulated, and the degree of down-regulation was greater the longer CtrA was depleted (Fig 2A). Thus, CtrA primarily functions as a positive regulator of transcription in *S*. *meliloti*. Among these positively regulated genes are many cell cycle regulators (i.e. *ctrA*, *divJ*, *divK*, *sciP*), motility genes (i.e. *mcp*, *che*, *flg*, *flaAB*, *fli)*, cell envelope components and several hypothetical proteins. The transcription of the genes encoding cell cycle regulators DivJ and DivK was significantly down-regulated after 2 hours of CtrA depletion in both the microarray and qPCR experiments (Fig 2B), strongly indicating that CtrA serves as a transcriptional activator of these two genes. Conversely, qPCR analysis revealed that expression of the cell division regulators *minC* and *minD* was strongly up-regulated in the absence of CtrA, indicating that CtrA is a transcriptional repressor of this operon (Fig 2B and 2C). The effect of CtrA depletion on the expression of *minCD* was particularly interesting because MinCD is a strong inhibitor of FtsZ ring formation and overexpression of MinCD in *S*. *meliloti* inhibits cell division [37]. Therefore, the increased expression of *minCD* may contribute to the block in cell division in cells depleted of CtrA.

### Analysis of CtrA binding sites by Chromatin Immunoprecipitation—Deep Sequencing (ChIP-Seq)

**Fig 3:**
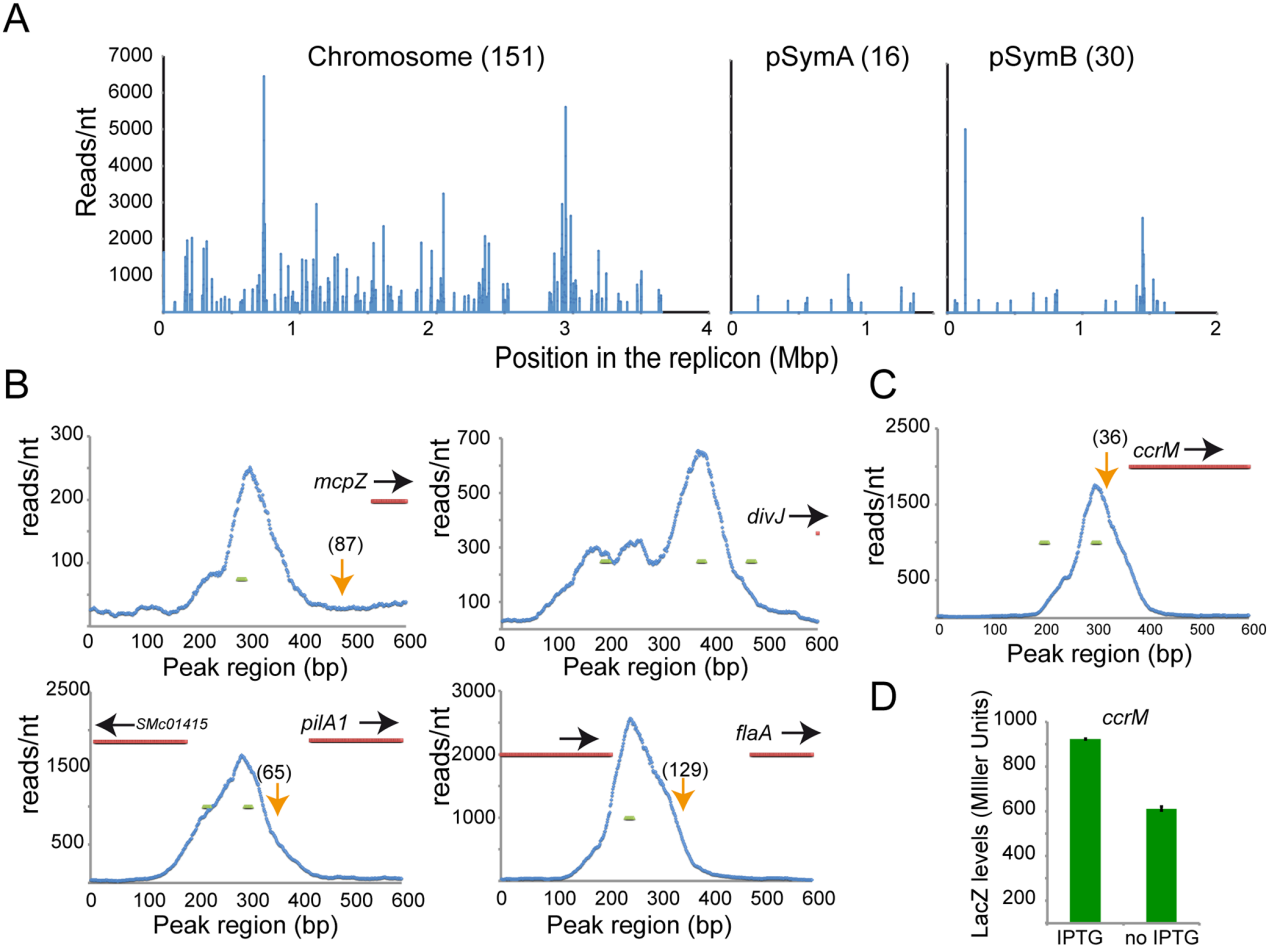
ChIP-Seq analysis reveals direct targets of CtrA. Genes directly regulated by CtrA. A. Representation of all CtrA binding sites in the three circular replicons of *S*. *meliloti* (here represented as linear starting from the origin of replication). B. Promoter region of several genes detected by microarrays. Transcriptional start sites, previously defined [39], are represented as orange arrows (numbers between brackets represent the distance form ATG). In blue the plot of reads per nucleotide measured by ChIP-Seq analysis in a 600bp long region including the beginning of the coding sequence (in red). Green lines represent predicted CtrA binding site [11]. C. ChIP-Seq of the *ccrM* promoter region. In blue the plot of reads per nucleotide measured by ChIP-Seq analysis. Green lines represent predicted CtrA binding site. D. Beta-galactosidase activity assay using a LacZ fusion of the *ccrM* promoter in cells (BM249) after depletion of CtrA (no IPTG) for two hours relative to control cells expressing CtrA (+IPTG). Error bars indicate standard error.

To discover the chromatin regions directly bound by CtrA in *S*. *meliloti*, we used ChIP-Seq analysis [38] (Materials and Methods). Starting from an exponential culture of *S*. *meliloti* cells, CtrA cross-linked DNA fragments of an average length of 300 base pairs were immunoprecipitated with antibodies raised against *C*. *crescentus* CtrA (CC3035), which can specifically bind *S*. *meliloti* CtrA [33]. Immunoprecipitated DNA was then deep-sequenced producing millions of ca. 50 nucleotide reads, which were mapped onto the *S*. *meliloti* genome to create a distribution of the number of reads per nucleotide (Fig 3A and S4 Table that shows the list of the 198 peaks across all three *S*. *meliloti* replicons). Correlating with our gene expression data, the chromosome contained most of the peaks (76%) while pSymB and pSymA contained only 15% and 10%, respectively. CtrA binding sites were mostly present in intergenic regions (79%), in line with a predicted regulatory role and with the microarray results [39] (see next section for more details).

We identified CtrA binding sites in the upstream regulatory regions of several key regulators of flagellum, pili, chemotaxis and cell cycle (Fig 3B) that were also identified by the microarray analysis. In particular, CtrA binding sites were detected in the promoter regions of *mcpZ*, *pilA1*, *flaA* and *divJ* and these binding sites overlapped with regions containing previously identified CtrA consensus sequences [11]. In order to confirm the direct transcriptional control of these genes by CtrA, their promoters were fused with *lacZ* and tested for their dependency on CtrA. Results showed that CtrA is a direct positive regulator of those genes (S4A Fig). Several genes were found to be bound by CtrA in the ChIP-Seq experiment (i.e. *rcdA*, *cpdR1*, *ccrM* and *pleC*), but not found to be differentially regulated in the CtrA depletion microarray. It is possible that these genes are direct transcriptional targets of CtrA, but the effect of CtrA depletion on their expression was missed due to the stringent cutoffs of the microarray data analysis (see Materials and Methods). These genes could also be subject to multiple levels of transcriptional regulation, which could compensate for the absence of CtrA in the system. Therefore, we used quantitative PCR and *lacZ* fusions to independently measure the effect of CtrA depletion on the expression of *rcdA*, *cpdR1*, *ccrM* and *pleC*. We found that *ccrM*, whose promoter was bound by CtrA in the ChIP-Seq analysis (Fig 3C), was significantly downregulated (Fig 3D) while *pleC*, *cpdR* and *rcdA* gave no significant changes consistent with the microarray analysis (S4 Fig).

### Identification of the direct and indirect regulons of *S*. *meliloti* CtrA

**Fig 4:**
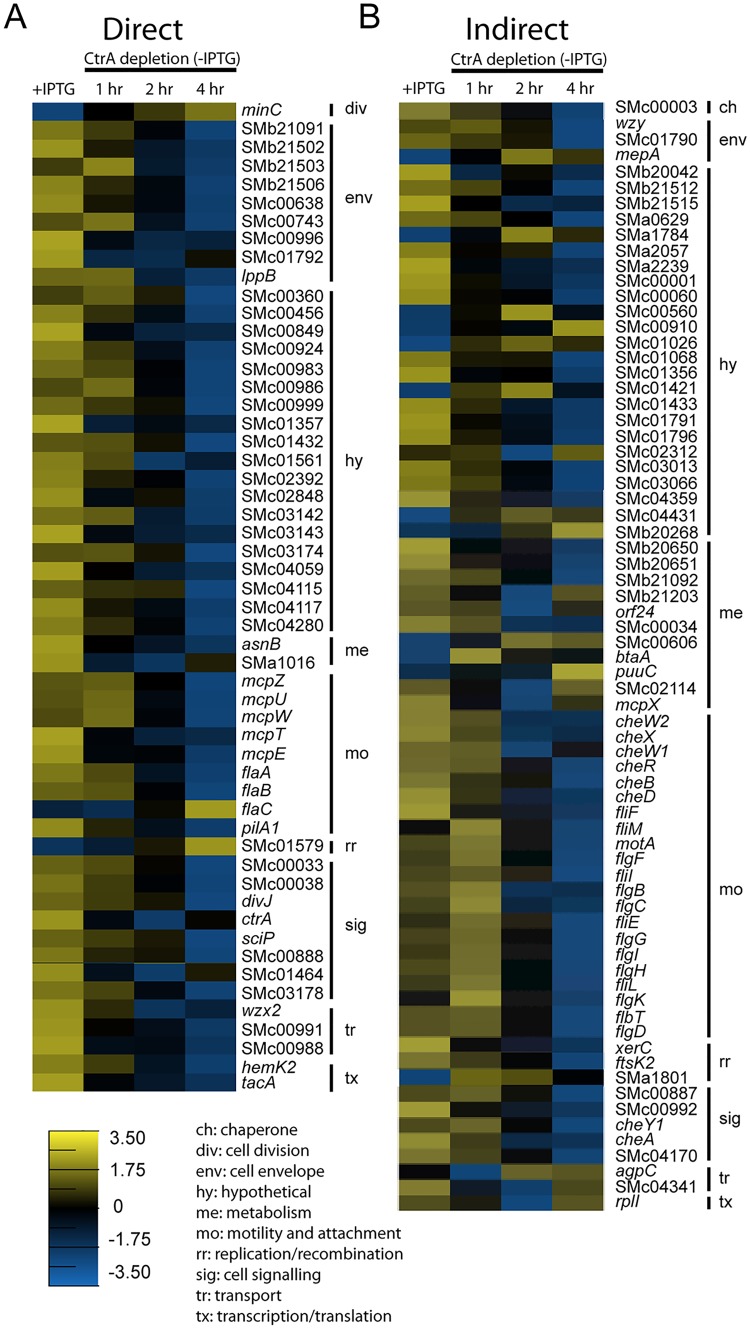
Expression profiles of direct and indirect targets of CtrA upon CtrA depletion. Expression profile of genes directly (A) and indirectly (B) controlled by CtrA. Shown are the average log2 expression levels for each gene in control cells (+IPTG) and the average log2 expression levels for each gene across each time point in cells depleted of CtrA (-IPTG). The scale for expression level is at the bottom of figure panel. Genes are grouped by functional classification explained in the legend on the bottom.

Combining our gene expression data from the CtrA depletion microarray and the CtrA ChIP-Seq analysis we were able to identify both the direct and indirect regulons of CtrA in *S*. *meliloti*. A total of 54 genes were both differentially expressed upon CtrA depletion and bound by CtrA in the ChIP-Seq analysis. These genes represent the experimentally determined direct regulon of *S*. *meliloti* CtrA and include genes encoding components of the cell envelope, motility and chemotaxis regulators, and signaling proteins (Fig 4A). The remaining 72 genes, which were differentially regulated upon CtrA depletion but not bound by CtrA in the ChIP-Seq experiment, comprise the indirect CtrA regulon in *S*. *meliloti* (Fig 4B). The proteins encoded by these genes are involved in many different cellular functions, with the most represented functional groups being motility and chemotaxis genes, metabolism genes and hypothetical genes (Fig 4B).

Taking advantage of previous analysis of transcription start sites (TSSs) in *S*. *meliloti* [39] we mapped the ChIP-seq peaks of the 54 genes of the direct regulon of CtrA with respect of their TSSs (S8 Table). Out of 54, 36 genes had a TSS downstream the ChIP-seq peak; also we mapped the predicted CtrA binding sites, defined as full or half sites as previously described [18], identifying CtrA predicted motifs in 47/54 promoters. This analysis suggests that ChIP-seq data are consistent with CtrA binding promoters of genes, whose expression change was detected by microarrays.

The majority of motility and chemotaxis genes are indirect targets of CtrA with the exception of the methyl-accepting chemotaxis genes *mcpT*, *mcpU*, *mcpW* and *mcpZ*; the flagellin genes *flaA*, *flaB* and *flaC*; and the pili gene *pilA1*. The genes encoding the primary flagellar apparatus of *S*. *meliloti* (i.e. *flgBCDH* and *fliEFIL*) were indirectly regulated by CtrA (Fig 4B), which is different from the direct regulation of these genes observed in *C*. *crescentus* [18]. This observation reinforces the hypothesis of a transcriptional regulatory hierarchy of *S*. *meliloti* flagellar and chemotaxis genes [40] and suggests that CtrA regulates the transcription of most of these genes with the exception of *fla* and *mcp* genes indirectly through secondary regulators. Because the promoter of the two-component response regulator *rem* contained a CtrA-binding motif and Rem regulates flagellar and chemotaxis genes, it was postulated that Rem could be the intermediate regulator [41]. However, *rem* was not differentially expressed in our CtrA depletion microarray experiment, nor was it directly bound by CtrA in our ChIP-Seq analysis, suggesting that CtrA likely acts through a different secondary regulator to control the transcription of flagellar and chemotaxis genes during the cell cycle (S4B Fig) [40,41].

In *C*. *crescentus*, CtrA directly activates the expression of antagonists of CtrA function (e.g. *cpdR*, *rcdA*, *clpP*, *sciP*, and *divK*) [11,27]. Interestingly, we found that only the direct regulation of *sciP* and *divJ* by CtrA was conserved in *S*. *meliloti*. SciP is an important modulator CtrA activity in *C*. *crescentus* swarmer cells [42,43]. Previous work has shown that transcription of *sciP* is cell cycle regulated in *S*. *meliloti* and, like in *C*. *crescentus*, activated only in the later stages of the cell cycle [40]. It is interesting that although *S*. *meliloti* does not exhibit the same level of morphological asymmetry as *C*. *crescentus*, the cell cycle and CtrA mediated regulation of SciP is conserved between the two species. Future studies on the function of SciP will provide insights on how this protein regulates CtrA function and the cell cycle. In contrast to *sciP*, the direct regulation of *rcdA* and *cpdR1*, which encode homologs of two proteins involved in regulation of CtrA proteolysis in *C*. *crescentus* by CtrA, was not conserved in *S*. *meliloti*. Although our ChIP-Seq analysis detected CtrA binding sites within the promoter regions of *rcdA* and *cpdR1*, no transcriptional effect was observed under CtrA depletion conditions (S4C Fig). Also unlike the *C*. *crescentus* paradigm, our data indicate that CtrA indirectly regulates *divK* transcription. The architecture of transcriptional control by CtrA on the DivK module in *S*. *meliloti* (*divJ* and *cbrA* are directly- while *divK* is indirectly-controlled) is very different from *C*. *crescentus* control by CtrA, which is only on *divK* [27]. Thereby this complex architecture gives an additional degree of freedom in the primary negative feedback loop that regulates CtrA function in *S*. *meliloti*.

We also found that CtrA directly regulates the expression of 19 hypothetical proteins and indirectly regulates the expression of 24 hypothetical proteins in *S*. *meliloti* (Fig 4B). Ortholog analysis using Microbes Online [44] revealed that orthologs of most of these hypothetical genes are not present in *C*. *crescentus*, except for SMc00910, SMc02312 and SMc02848 (CC0705, CC2340 and CC3721, respectively in *C*. *crescentus*). However these genes are not regulated by CtrA in *C*. *crescentus* [18]. It will be especially interesting to determine the role of these genes in the cell cycle and physiology of *S*. *meliloti* in future work.

Our data also revealed that *minC* and *minD* are the only characterized cell division genes directly controlled by CtrA in *S*. *meliloti* (Fig 4A). The analysis revealed that CtrA is a repressor of *minCD* transcription, strongly suggesting that CtrA contributes to the expression pattern of *minCD* observed during the *S*. *meliloti* cell cycle [30]. In *S*. *meliloti* and many other bacteria, MinC and MinD repress cell division by inhibiting FtsZ polymerization and Z-ring formation [45]. In *S*. *meliloti*, CtrA may promote FtsZ polymerization and cell division by repressing *minCD* in predivisional cells. Placement of the Z-ring is not regulated by the Min system in *C*. *crescentus* and instead CtrA directly controls the transcription of *ftsQA* [18,46,47]. Thus CtrA regulation of cell division in *S*. *meliloti* may have specifically evolved to control FtsZ polymerization indirectly through the Min system.

In order to test this possibility we used M12 phage to transduce a *min* operon deletion cassette [37] into the *ctrA* depletion strain (BM249). Since the *min* operon is dispensable for proper growth of *S*. *meliloti* [37] this strain (EB1441) was viable when grown in 1mM IPTG. We hypothesized that deletion of the *min* cassette may partially rescue the arrested cell division phenotype of the *ctrA* depletion strain, however without supplemental IPTG EB1441 was unable to divide normally (no colonies were recovered after 7 days transducing the *min*::Spec without IPTG). However, when EB1441 was grown in 1mM IPTG and then transferred in to medium lacking IPTG (CtrA-loss of function) EB1441, similar cell division defects as the *ctrA* depletion strain were observed (S7 Fig). This result indicates that, although the overexpressed *min* system may contribute to the cell division defect of the CtrA depletion, it is not the only mechanism involved. For example, nucleoid occlusion may serve as a potent mechanism to block cell division, especially due to the severe amplification of the genome in CtrA depleted cells [48]. Moreover, CtrA may act on cell division through unknown genes of its regulon that may have an important role in *S*. *meliloti* cell division control.

### Regulated proteolysis, but not transcriptional autoregulation is an important mechanism of CtrA regulation in *S*. *meliloti*

**Fig 5:**
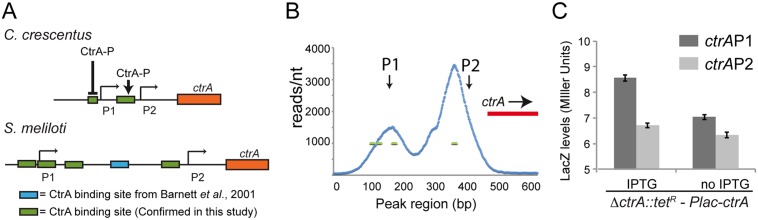
Structure of the *ctrA* promoter in *S. meliloti*. A. Promoters of *ctrA* in *C*. *crescentus* and *S*. *meliloti*. Both promoters have two transcriptional sites (P1 and P2). In *C*. *crescentus* P2 is activated by CtrA-P while P1 is activated by GcrA and repressed by CtrA-P [20,64,72]. In *S*. *meliloti* the P1 and P2 transcriptional start sites have been previously defined by primer extension [28]. ChIP-Seq results identified 4 binding sites of CtrA in *S*. *meliloti* upstream P1 and P2 while previously a fifth one was discovered by DNase I footprinting [28]. The presence of CtrA binding sites suggests a potential control of transcription by CtrA; B. Details of the ChIP-Seq using antibodies against CtrA (blue) of the *ctrA* promoter region. C. Promoters P1 and P2 were fused to *lacZ* measuring the beta—galactosidase activity depleting CtrA for 4 hours.

**Fig 6:**
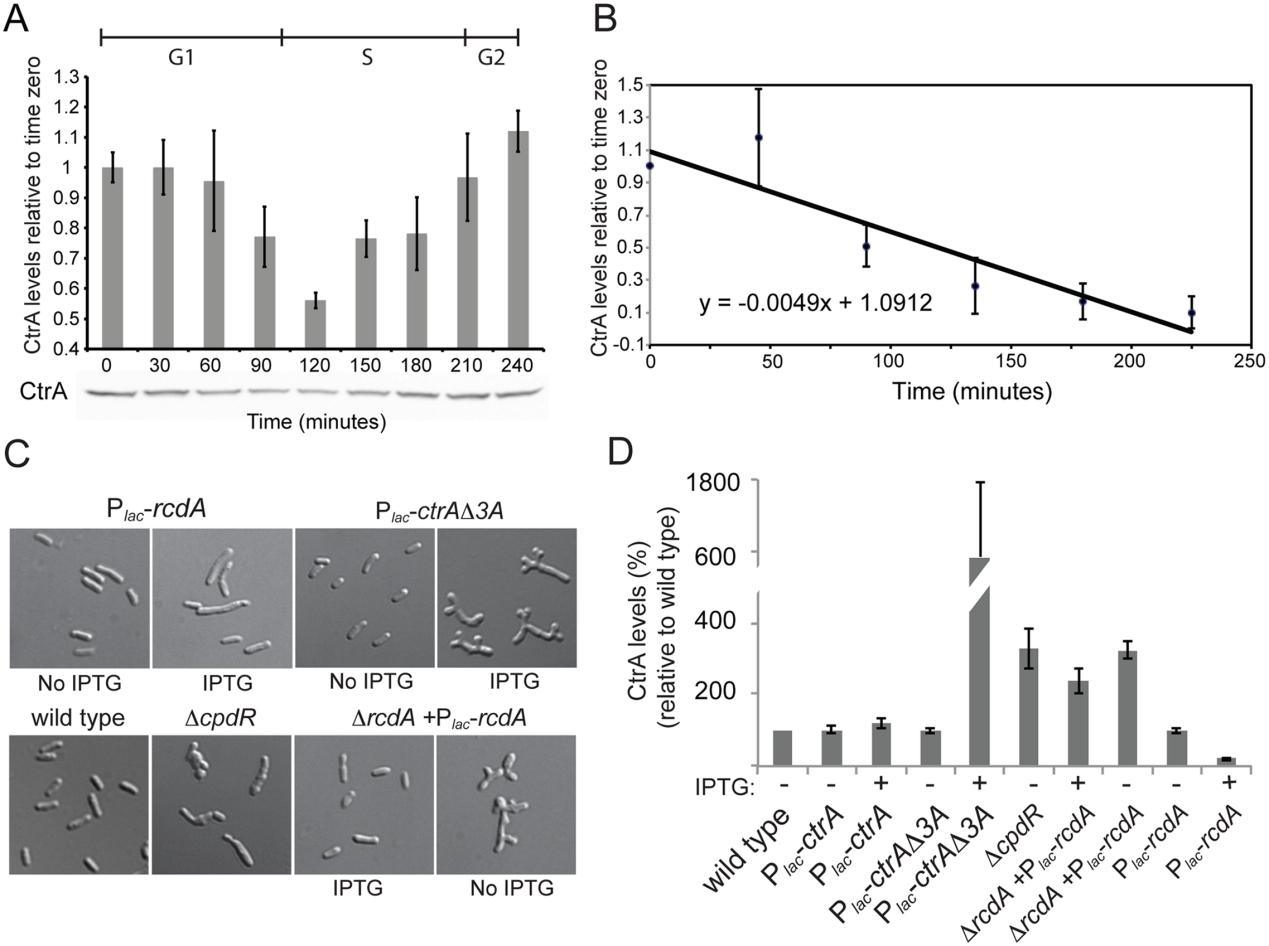
Proteolysis of CtrA is essential in *S*. *meliloti* and requires at least CpdR, RcdA and the last three amino acids of CtrA. A. CtrA protein level changes during the cell cycle with a minimum around 120 min that corresponds to the G1-S transition [40]. Cells were synchronized and samples were collected every 30 minutes. CtrA antibodies were used to detect the protein level, protein levels were normalized for cell number and error bars represent standard error; B. Pulse-chase experiment of showing decrease over time of radiolabeled CtrA in *S*. *meliloti* cells. Values are averages from three separate experiments and the error bars represent standard deviation. C. Morphology of CtrA degradation defective mutants. CpdR^-^ [29], although barely vital, shows compromised cell morphology. Cell depleted of RcdA for 7 hours also have altered morphology. Over-expression of *rcdA* for 7 hours causes cell elongation and division defects (Fig 1C). Overexpression of a stable version of CtrA (lacking the last three amino acids) for 7 hours causes altered cell morphology similar to that of the RcdA depletion strain. D. CtrA protein levels (% of CtrA in wild type cells) in the genetic backgrounds described in the panel C. Cell lysates were normalized for protein content, error bars represent standard error of three different replicates.

Bioinformatic prediction [11], our ChIP-Seq experiment and DNase I footprinting analysis [28] identified several CtrA binding sites in both the P1 and P2 promoter region of *ctrA* (Fig 5B). To determine if the *ctrA* auto-regulation observed in *C*. *crescentus* was conserved in *S*. *meliloti*, the *lacZ* fusions of *ctrA* P1 and P2 were tested in the *ctrA* depletion strain (Fig 5A). Upon *ctrA* depletion, both P1 and P2 showed mild changes of expression compared to non-depleted cells suggesting that, differently from *C*. *crescentus*, *S*. *meliloti* CtrA does not strongly activate its own promoters (Fig 5C). However, we observed a mild but significant decrease of CtrA expression by P1 in CtrA depleted cells compared to cells supplemented with IPTG, suggesting that CtrA may positively regulate its own transcription at P1, but CtrA depletion had no significant effect on transcription from P2 (Fig 6A). Thus, contrary to *C*. *crescentus* where P2 is strongly regulated by CtrA, *S*. *meliloti* CtrA only weakly activates its P1 promoter. Therefore, it is likely that other factors are involved in the transcriptional regulation of CtrA in *S*. *meliloti*, and future work will focus on identification of these regulators.

Another important mode of CtrA regulation in *C*. *crescentus* is regulated proteolysis by the ClpXP protease [49]. It is unknown whether CtrA activity in *S*. *meliloti* is subject to a similar posttranslational regulatory network as it is in *C*. *crescentus*. The regulated proteolysis of CtrA is especially interesting in the context of *S*. *meliloti* symbiosis since regulated proteolysis represents an efficient mechanism by which CtrA could be eliminated from differentiating bacteroids [33–35]. To shed light on the mechanism of CtrA regulation in *S*. *meliloti* and to understand how CtrA levels may be downregulated in the bacteroid during symbiosis, we first checked if CtrA protein levels dynamically changed over the cell cycle as in *C*. *crescentus*. *S*. *meliloti* cells were synchronized as previously described [30] and CtrA levels were measured over the course of the cell cycle by immunoblotting (Fig 6B). We observed a drop in CtrA levels around the G1-S transition in a similar fashion as in *C*. *crescentus*. Although *S*. *meliloti* CtrA contains a putative C-terminal ClpX targeting tag [22], it has not been demonstrated that *S*. *meliloti* CtrA is subject to the same cell cycle regulated proteolysis as observed in *C*. *crescentus* [21,22]. To test whether *S*. *meliloti* CtrA may be regulated by proteolysis during the cell cycle, we determined the half-life of CtrA using pulse/chase analysis. We pulsed mid-log phase cultures with ^35^S-labeled methionine and cysteine, chased with unlabeled methionine and cysteine, and pulled down the *S*. *meliloti* CtrA protein using a *C*. *crescentus* CtrA polyclonal antibody [50]. We directly determined the half-life of ^35^S labeled *S*. *meliloti* CtrA to be 141 minutes during a ~220 minute cell cycle (Fig 6B), as compared to the 53 minute half-life of *C*. *crescentus* CtrA in unsynchronized culture during a 160 minute cell cycle [19,20]. These results suggest that CtrA in *S*. *meliloti* is subjected to active proteolysis although it is difficult to directly compare half lives between the two species due to the likely differing ratios of G1-arrested and dividing cells in unsynchronized cultures.

We next wanted to test the effect of inhibiting CtrA proteolysis in *S*. *meliloti* to assess its importance in cell cycle regulation. In *C*. *crescentus*, both *ctrADD*, where the C-terminal alanine residues were converted to aspartate residues, and *ctrAΔ3*, where the last three amino acids were deleted, produce stabilized versions of CtrA [21,22]. We first attempted to introduce these two alleles of *ctrA* into *S*. *meliloti* to assess the effects of CtrA stabilization. Introducing both the *ctrADD* and *ctrAΔ3* allele to *S*. *meliloti* via expression from a medium copy plasmid or by direct integration into the native *ctrA* locus by the *sacB* suicide method yielded no transconjugants, suggesting that the stable derivatives of CtrA are lethal in *S*. *meliloti*. To confirm the lethality of these putative non-degradable CtrA alleles in *S*. *meliloti*, *ctrAΔ*3 was cloned into an inducible pSRK-Km derivative plasmid under a P_*lac*_ promoter and mated into *S*. *meliloti*. Upon induction of CtrAΔ3 expression loss of viability was observed (S5 Fig), with dramatic cell cycle morphological defects after 6 hours of induction (Fig 6C) that corresponded with increased CtrA protein levels (Fig 6D). Our results hence suggest that proper proteolytic regulation of CtrA levels are essential in *S*. *meliloti*.

If proteolysis of CtrA is essential in *S*. *meliloti*, mutations in genes coding for putative co-factors of proteolysis should lead to severe phenotypes and result in an abnormal increase of CtrA levels. CpdR and RcdA both play a crucial role in controlling CtrA proteolysis in *C*. *crescentus* [26,51]. CpdR was previously shown to have physiologically important roles in *S*. *meliloti* but its direct link to CtrA proteolysis was not examined [29]. Immunoblotting revealed that CtrA levels were *ca*. 3 times higher in a *cpdR1* mutant relative to wild type cells (Fig 6D), consistent with a role for CpdR1 in promoting CtrA degradation in *S*. *meliloti* and suggesting that the striking phenotype of a *cpdR1* mutant might be due, in part, to elevated CtrA levels (Fig 6C) [29]. However, when we attempted to construct a *ΔrcdA* derivative of *S*. *meliloti*, we could only do so in the presence of plasmid bearing the *rcdA* gene expressed under its native promoter or P_*lac*_ (S5 Table). We were also unable to transduce the *Δrcd*A into wild-type *S*. *meliloti* in the absence of ectopically expressed *rcdA* (S5 Table), further indicating that *rcdA* is an essential gene in *S*. *meliloti*.

To gain insights onto how RcdA affects cell viability and CtrA protein levels, we used the previously described pSRK system to create a *rcdA* depletion strain. Cells depleted of RcdA were elongated and branched with irregular and enlarged bodies, a phenotype reminiscent of cells lacking *cpdR1* and cells expressing non-degradeable CtrAΔ3 protein [29] (Fig 6C). We next examined the effect of varying RcdA levels on CtrA levels by immunoblotting. CtrA levels were elevated in the *rcdA* depletion and were reduced under conditions of *rcdA* overexpression (Fig 6D). Collectively, these results strongly suggest that, as in *C*. *crescentus*, RcdA, CpdR and the last three amino acids of CtrA are all required for correct proteolysis of CtrA. Differently from *Caulobacter*, proteolysis plays an essential role in *S*. *meliloti* as any impairement of CtrA proteolysis causes a cell cycle arrest.

## Discussion

**Fig 7:**
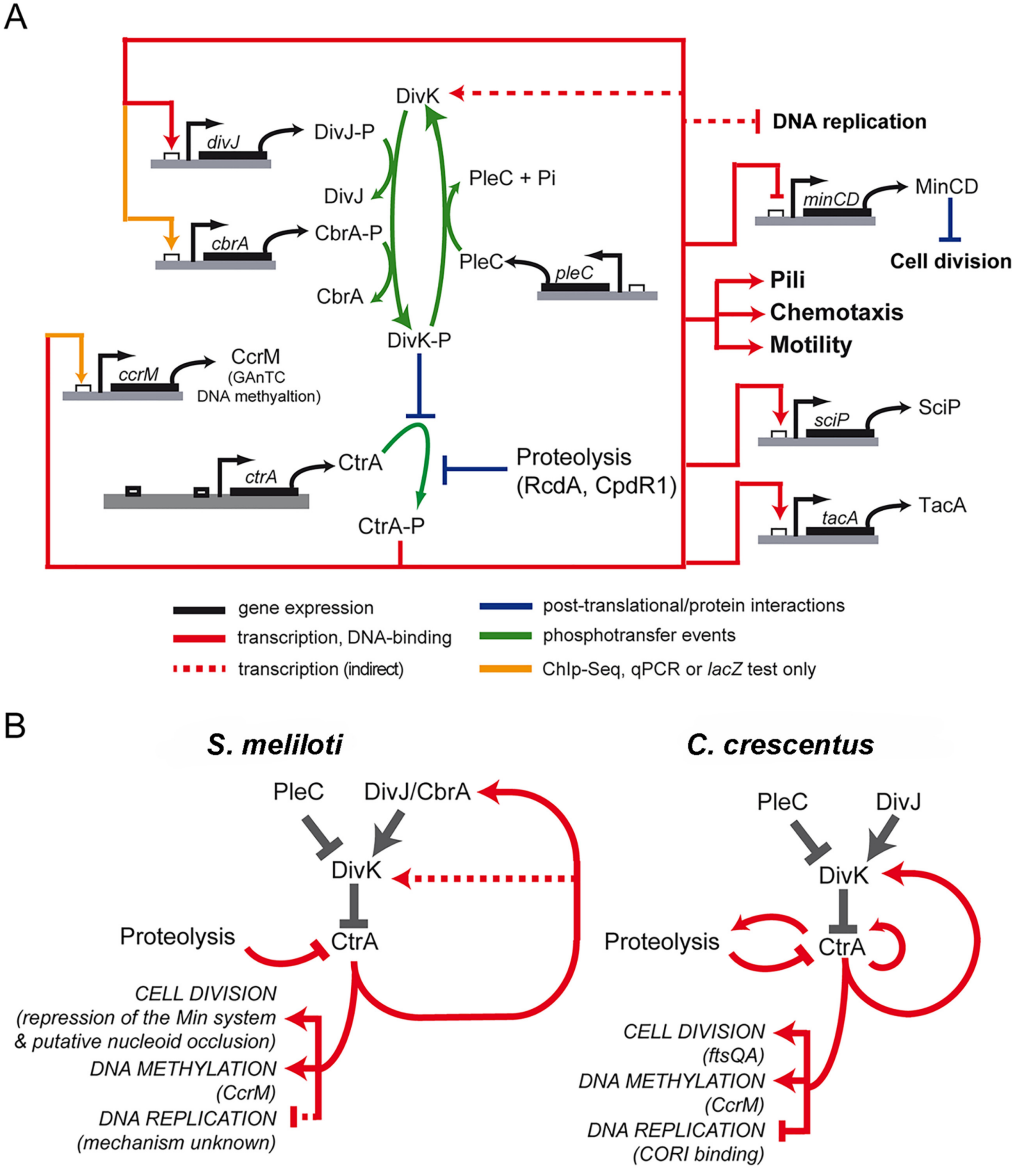
Model of CtrA network in *S*. *meliloti*. A. Scheme of genes regulated by CtrA. As reported in the legend two kinds of connections are reported: in red those confirmed by both ChIP-Seq and microarray and in yellow those not detected by microarrays but confirmed by other techniques. Phosphorylation of CtrA is essential [28] and the roles of DivJ, PLeC and CbrA have been previously described [33]. Despite the representation here, there is no indication of the preferred form of CtrA subjected to proteolysis. CtrA working on the promoters of genes is a simplification to represent of the direct effect of CtrA on transcription of the gene. B. Comparison between the circuit regulating cell cycle in *S*. *meliloti* and *C*. *crescentus*. Although the two organisms share the same logic of cell cycle regulatory circuit, differences in the factors connected and involved in the regulation of specific functions are present.

In this work we present significant progress towards the understanding of *S*. *meliloti* cell cycle regulation and differentiation in bacteroids, specifically involving the conserved master cell cycle regulator CtrA (Fig 7A). We showed that CtrA-deprived cells are unable to divide, exhibit cell elongation, branching and a sharp increase in DNA content. These combined phenotypes result in a clear loss of viability. Through microarray-based gene expression analysis coupled with ChIP-Seq analysis we defined the direct and indirect regulons of CtrA and discovered that, in agreement with the loss of function phenotypes, CtrA acts as a transcriptional regulator controlling essential functions that are required for proper cell cycle progression, such as cell division, chromosome methylation, motility and cell envelope biogenesis. Similarly to *C*. *crescentus*, CtrA is subjected to several regulatory mechanisms. We showed that the concentration of CtrA drops at the moment of the G1 to S transition similarly to *C*. *crescentus* [22]. Taken together with the striking genome amplification observed during CtrA depletion, these data suggest that CtrA is a repressor of DNA replication initiation that must be inactivated at the G1-S transition and perhaps during bacteroid differentiation. Furthermore, perturbations of the last three C-terminal residues of CtrA results in a marked accumulation of CtrA protein, suggesting that like in *C*. *crescentus*, *S*. *meliloti* CtrA levels are regulated by proteolysis, likely by the ClpXP protease [49]. We also found that levels of CtrA are higher in mutants lacking functional CpdR and RcdA, which are key regulators of CtrA proteolysis in *C*. *crescentus* [26,51]. Surprisingly, however, unlike *C*. *crescentus*, the disruption of CtrA proteolysis is lethal in *S*. *meliloti* as both deletion of *rcdA* and expression of *ctrAΔ3* are lethal.

The comparison between the *S*. *meliloti* CtrA circuit architecture with *C*. *crescentus* helped us to understand several general principles of the alphaproteobacterial cell cycle (Fig 7B). First, CtrA plays a crucial role in directly regulating genes involved in motility, chemotaxis and cell division. Motility was identified as an ancestral functional of this regulator in *Magnetospirillum magnetotacticum* and other alphaproteobacteria [13]. In most cases, however, the regulation of these similar processes is achieved through the control of different target genes. For example, CtrA controls cell division through *ftsAQ* in *C*. *crescentus*, while in *S*. *meliloti* this control is likely carried out by other means, such as CtrA repression of the Min system together with other unknown mechanisms, including for example nucleoid occlusion. This suggests that the CtrA regulon has changed significantly during the evolution of alphaproteobacteria and that different regulatory networks have evolved in the same phylogenetic group. A second general feature of the CtrA circuit in alphaproteobacteria concerns the link between CtrA and the DivK/DivJ module, which directly controls the phosphorylation status and indirectly controls the stability of CtrA. In both *C*. *crescentus* and *S*. *meliloti*, this module is controlled by CtrA via transcriptional regulation. In *C*. *crescentus* this important feedback loop is routed through the *divK* promoter, which is directly activated by CtrA [20]. In *S*. *meliloti*, *divK* transcription is only indirectly affected by CtrA depletion, and instead, CtrA feedback regulation of this module happens directly through the transcriptional activation of *divJ* by CtrA (Fig 7). The gene encoding the DivJ cognate kinase, *cbrA*, may also regulated directly by CtrA as its promoter was bound in the CtrA ChIP-Seq experiment, but significant differential expression of *cbrA* under conditions of CtrA depletion was only detected by *lacZ*-fusions and not by microarrays (S6 Fig). Previous work has found that altering the native promoter of *divK* in *C*. *crescentus* causes severe cell cycle defects [27], while in *S*. *meliloti* altering *divJ* transcription leads to an uncoordinated progression through the cell cycle, in particular, its overexpression causes a G2 block [33]. These and other interesting divergences in the wiring of the *S*. *meliloti* and *C*. *crescentus* cell cycle network are just the tip of the iceberg in uncovering how alphaproteobacteria have evolved species-specific wiring of these highly conserved cell cycle components to fit their unique lifestyles and diverse cellular differentiation programs.

Perhaps most importantly towards the understanding of symbiotic interactions with legume hosts, our findings provide insight into how *S*. *meliloti* cell cycle regulation may be altered during bacteroid differentiation to produce the bacteroid phenotypes of cell elongation and endoreduplication. Within the host nodule cells, *S*. *meliloti* is exposed to a microaerobic environment, which activates the FixJ/FixL two-component system. Activation of this system elicits a significant transcriptional response including expression of genes coding for the nitrogen fixation machinery [52–55]. Another important trigger of the bacteroid differentiation process is a large class of nodule-specific cysteine rich (NCR) peptides produced by the host legume [4,7,35]. The specific cellular targets of these peptides are unknown, although recent work has provided strong evidence that at least one of these peptides, NCR247, targets diverse cellular processes in *S*. *meliloti* [7,35,56], including the cell cycle. Furthermore, treatment of *S*. *meliloti* with NCR247 directly affects the transcription of CtrA and many other genes included in the direct and indirect CtrA regulons established in this work [35]. The observations that *ctrA* transcriptional downregulation coincides with bacteroid differentiation within the nodule [34], that CtrA is largely absent from mature bacteroids [33], and that an NCR peptide specifically perturbs the CtrA transcriptional regulon [35] all point to CtrA as an important regulatory node through which the legume host manipulates the bacterial cell cycle. Interestingly the dissection of gene expression changes in the differentiation region of the nodule revealed that the formation of bacteroids is associated increased concentration of *rcdA* transcript [34], which correlates with our observation that *rcdA* overexpression enhances CtrA degradation (Fig 6D). This work provides the first global overview of the CtrA cell cycle regulon in *S*. *meliloti*, which it will facilitate the exploration how NCR peptides and other plant factors affect the function of CtrA and other important cell cycle regulators to drive bacteroid differentiation in *S*. *meliloti*.

## Materials and Methods

### Bacterial strains and growth conditions

The bacterial strains and plasmids used in this study are listed in S6 Table. *E*. *coli* strains were grown in liquid or solid lysogeny broth (LB) (Sigma Aldrich) [57] at 37°C supplemented with appropriate antibiotics: kanamycin (50 μg ml^-1^ in broth and agar), tetracycline (10 μg ml^-1^ in broth and agar) and gentamycin (15 μg ml^-1^ in liquid broth, 20 μg ml^-1^ in agar). *S*. *meliloti* strains were grown in broth or agar TY [58] supplemented when necessary with kanamycin (200 μg ml^-1^ in broth and agar), streptomycin (500 μg ml^-1^ in broth and agar), tetracycline (1 μg ml^-1^ in liquid broth, 2 μg ml^-1^ in agar), spectinomycin (50 μg ml^-1^ in broth and agar) and gentamycin (20 μg ml^-1^ in broth and agar). For negative selection 1% sucrose was added to agar plates. Depletion conditions were tested growing cells to mid-log phase (OD_600_ = 0.6) in media containing IPTG (1mM for *ctrA*, and 80μM for *rcdA*), and then resuspended at OD_600_ = 0.1/0.2, after 2 washes, in media lacking IPTG. Synchronization experiments were performed as described previously [40].

### Strain constructions and general techniques

Deletion mutants of *ctrA* and *rcdA* were constructed by two-step recombination of deletion cassettes, conducted as previously described using derivatives of the integrative plasmid pNPTS138 [33]. The first integration of the plasmid has been done by conjugation; 1 × 10^9^ *S*. *meliloti* and 0.5 × 10^9^ *E*. *coli* S17-1 cells [59] were used and incubated 24 h at 30°C. As simple deletion of the gene was not possible the procedure was performed in strain carrying a complementation plasmid. Deletions were verified by PCR using primers external to the area of recombination (see primers in S7 Table).

Complementation plasmids (pMR10 derivatives) [60], pSRK [36] derivatives (to study depletion and overexpression conditions) and pRKlac290 [61] derivatives (P-*lacZ* transcriptional reporter strains) were introduced by electroporation [62].

For transduction, M12 phage [63] and bacteria (in LB containing 2.5 mM CaCl_2_ and 2.5 mM MgSO_4_) were mixed to give a multiplicity of infection 1/2 (phage per cell). The mixture was incubated at 30°C for 30 min and subsequently plated on LB plates with the appropriate antibiotics [33].

For the efficiency-of-plating (EOP) assays showed in S5 Fig, cultures were grown to mid exponential phase (OD_600_ ≈ 0.55) in TY medium. Each sample was serially diluted up to 10^-6^ in TY, and spread onto TY agar with and without IPTG (1 mM). After 3 to 5 days of growth at 30°C, the number of colonies was determined. The average and standard deviation for each strain were derived from three independent cultures.

B-galactosidase assays and western blots were performed as previously described [64].

More details on cloning are in supporting S1 Text.

### Pulse-chase analysis of CtrA in *S*. *meliloti*

Pulse-chase analysis was performed as described in (Chen, Sabio and Long 2008). *S*. *meliloti* was grown in SMM media to mid-log phase (OD_600_ ~0.6–0.7). Cultures were pulsed with 20uCi of ^35^[S]met and cys EasyTagEXPRESS protein labeling mix (Perkin Elmer) and chased with 100x chase solution (0.4% methionine, 0.3% cysteine). Time points were taken and pelleted at 10,000xg for 3 minutes every 45 minutes including 0 minutes after chase. Each time point was resuspended in 10x TEN (100mM Tris pH8, 10mM EDTA pH8, 0.25 NaN_3_), pelleted and resuspended in 1X TEN and put on ice. Once all time points were collected each sample was pelleted again and then resuspended in 50uL TES (10mM Tris pH8, 1mM EDTA pH8, 1% SDS). All samples were incubated at 100C for 10 minutes, 1mL of IP buffer (50mM Tris, pH7.5, 150mM NaCl, 1% Triton X-100) plus Sigma protease inhibitors (1:250) were added to each tube the pellet was resuspended and the samples were pelleted again. The supernatant was pre-cleared by incubation with 25mL of 50% slurry Protein A agarose (Pierce) at 4C for 20 minutes. 750uL of supernatant was transferred to a new tube and 1uL of CtrA antibody (*C*. *crescentus* CtrA antibody R308 gift of Michael Laub) and 25uL of 50% slurry Protein A agarose and rotated for 4 hours at 4C. Samples were washed 3x with IP buffer and 1x with IP buffer without Triton. Samples were resuspended in 12uL of 2x sample buffer and stored at -80C. Samples were run on 4–20% Tris-HCl gel and the gel was dried. Dried gels were exposed Amersham Biosciences Storage Phosphor Screen and developed using a Typhoon imager.

### Chromatin Immunoprecipitation (ChIp)

Mid-log phase cells (80 ml, OD_600_ of 0.6) were cross-linked in 10 mM sodium phosphate (pH 7.6) and 1% formaldehyde at room temperature for 10 min and on ice for 30 min thereafter, washed thrice in phosphate buffered saline (PBS) and lysed with lysozyme 2.2 mg ml^-1^ in TES (Tris-HCl 10 mM pH 7.5, EDTA 1 mM, NaCl 100 mM). Lysates (Final volume 1ml) were sonicated (Branson Digital Sonicator 450, Branson Sonic Power. Co., www.bransonic.com/) on ice using 10 bursts of 20 sec (50% duty) at 30% amplitude to shear DNA fragments to an average length of 0.3–0.5 kbp and cleared by centrifugation at 14,000 rpm for 2 min at 4°C. Lysates were normalized by protein content by measuring the absorbance at 280 nm; ca. 7.5 mg of protein was diluted in 1 mL of ChIP buffer (0.01% SDS, 1.1% Triton X-100, 1.2 mM EDTA, 16.7 mM Tris-HCl [pH 8.1], 167 mM NaCl plus protease inhibitors (Roche, www.roche.com/) and pre-cleared with 80 μL of protein-A agarose (Roche, www.roche.com/) and 100 μg BSA. Polyclonal antibodies to CtrA [33] were added to the remains of the supernatant (1:1,000 dilution), incubated overnight at 4°C with 80 μL of protein-A agarose beads pre-saturated with BSA, washed once with low salt buffer (0.1% SDS, 1% Triton X-100, 2 mM EDTA, 20 mM Tris-HCl (pH 8.1), 150 mM NaCl), high salt buffer (0.1% SDS, 1% Triton X-100, 2 mM EDTA, 20 mM Tris-HCl (pH 8.1), 500 mM NaCl) and LiCl buffer (0.25 M LiCl, 1% NP-40, 1% sodium deoxycholate, 1 mM EDTA, 10 mM Tris-HCl (pH 8.1) and twice with TE buffer (10 mM Tris-HCl (pH 8.1) and 1 mM EDTA). The protein•DNA complexes were eluted in 500 μL freshly prepared elution buffer (1% SDS, 0.1 M NaHCO_3_), supplemented with NaCl to a final concentration of 300 mM and incubated overnight at 65°C to reverse the crosslinks. The samples were treated with 2 μg of Proteinase K for 2 h at 45°C in 40 mM EDTA and 40 mM Tris-HCl (pH 6.5). DNA was extracted using QIAgen minelute kit and resuspended in 30 μl of Elution Buffer. ChIp DNA sequencing was performed using Illumina MySeq and analyzed as previously described [64]. Raw fastq data are available upon request.

### Microscopy

*S*. *meliloti* cells were grown to mid-log phase, fixed in 70% ethanol, washed and concentrated with GTE (50 mM glucose, 10 mM EDTA, 20 mM Tris,pH 7.5). Bacteroids were extracted as previously described [33]. Samples were stained with Hoechst 33324 (Cf 5μg/ml) and Propidium iodide (Cf 2μg/ml) for 30 minutes at RT. Samples were deposited on microscope slides coated with 0.1% poly-L-lysine. Images were processed with ImageJ [65].

### Microarray hybridization and analysis

Exponential phase cells grown in the presence of 1mM IPTG were pelleted by centrifugation washed twice with 0.85% saline solution, and split into two cultures that contained either 1mM IPTG or no IPTG (CtrA depletion). RNA was isolated from triplicate samples of t = 0 *S*. *meliloti* cells as well as from +IPTG (control) and—IPTG (*ctrA* depleted) 1, 2, 4 and 6 hours after depletion were converted to cDNA and hybridized to custom Agilent microarrays containing 6046 *S*. *melioti* ORF (GPL18182). To determine which *S*. *meliloti* genes may be transcriptionally regulated by CtrA, the log_2_ fold change (logFC) of expression between average triplicate-IPTG and +IPTG samples at 1, 2, and 4 hours were calculated using the *limma* package in R. The 6-hours time point, although highly correlative with the earlier time points, was excluded from the analysis due to a lack of replicate samples. RNA isolation, cDNA synthesis and labeling, and microarray hybridization are as described [40]. Only one channel was used for hybridization.

Data were normalized as previously described [35]. Normalized microarray data of IPTG-treated and non-treated (CtrA-depletion) samples were directly compared by using the Limma package in R [66,67]. A linear model was fitted to the normalized log2 values for each gene at the 1-, 2,- and 4-hour time points and used to generate estimated coefficients and standard errors for the compared samples. Moderated t-statistics, moderated F-statistics and log-odds of differential expression using an empirical Bayes approach were applied to the parameter estimates and standard errors from the linear models for each probe. P-value adjustment for multiple testing was performed using the Benjamini-Hochberg false discovery rate procedure. Genes identified as differentially expressed had logFC values ≥ 1.0 or ≤ –1.0 and an adjusted p value of ≤ 0.05.

For heat map generation, the replicate-average log2 expression values for the 126 differentially expressed gene identified above were row normalized across the time points as described [35]. Normalized values were then clustered by using Gene Cluster 3.0 and the city block similarity metric with complete linkage clustering. For the heat map of direct and indirect targets of CtrA, the average log2 expression for each gene in +IPTG samples (1-, 2- and 4-hour) and the replicate-average log2 expression values for a gene at each time point was row normalized and clustered as described above. The microarray data discussed in this publication have been deposited in NCBI's Gene Expression Omnibus [68,69] and are accessible through GEO Series accession number GSE68218 (http://www.ncbi.nlm.nih.gov/geo/query/acc.cgi?acc=GSE68218).

### FACS analysis

Flow cytometric analysis of DNA content in *S*. *meliloti* cells was performed as previously described [40].

### Quantitative RT-PCR

The 2^-ΔΔCT^ method was used to determine the expression level of indicated genes [70]. The fold change in gene expression in CtrA-depleted cells was plotted relative to gene expression in CtrA-replete cells. The expression level of the control gene *smc00128* [71] was used to normalize expression data in cells replete with CtrA and cells lacking CtrA. Oligonucleotide primers are available upon request.

## Supporting Information

S1 Text Cloning and real time PCR methods. (PDF)

S1 Table Transduction of *tetR* deletion of *ctrA* in different genetic backgrounds. (PDF)

S2 Table List of all genes in all conditions logFC and log2 expression values. (XLSX)

S3 Table Differentially expressed *S*. *meliloti* genes under CtrA depletion conditions. See S1 Text for details. (XLSX)

S4 Table ChIP-Seq best hits. (PDF)

S5 Table Transduction of *tetR* deletion of *rcdA* in different genetic backgrounds. (PDF)

S6 Table Strains and plasmids used in this work. (PDF)

S7 Table Primers used in this work. (PDF)

S8 Table TTSs mapping in the 54 direct targets of CtrA. (PDF)

S1 Fig Western blotting of CtrA depleted cells (BM249) in comparison with wild type. (PDF)

S2 Fig Nomarski (DIC) and fluorescence microscopy of bacteroids (isolated from active nitrogen fixing nodules), wild type cells and Δ*ctrA* + P_lac_-*ctrA* without (6h depletion) and with 1mM IPTG stained with Hoechst 33342 and propidium iodide (PI). “Heat-treated” indicates 10-min treatment at 70°C, as in Mergaert et al., 2006 (Scale bar = 5 μm).
(PDF)

S3 Fig qPCR of genetic markers of each replicon of *S*. *meliloti*. (PDF)

S4 Fig **A. β-galactosidase assays of genes of Fig 3B B.** Fold change in *rem* expression in cells after depletion of CtrA (-I, IPTG) for two hours relative to control cells expressing CtrA (+I). Expression of *rem* was normalized to the expression of the control gene *smc00128*. Shown are data from a representative biological replicate. Error bars indicate standard error. C. Fold change in *cpdR1* and *rcdA* expression in cells after depletion of CtrA (-I, IPTG) for four hours relative to control cells expressing CtrA (+I). Expression of *cpdR1* and *rcdA* was normalized to the expression of the control gene *smc00128*. Data are shown from a representative biological replicate. Error bars indicate standard error.
(PDF)

S5 Fig Overxpression of *ctrA*Δ3A is not tolerated by cells. Triplicate cultures of Rm1021 + P_lac_ (EB261) and Rm1021 + P_lac_ *ctrA* (EB776) were assayed for growth inhibition by CtrA overexpression plating in presence (grey bars) or not (black bars) of 1mM IPTG. The number of colonies was determined after 4 to 5 days of growth without IPTG at 30°C (further incubation of the plates did not result in the appearance of additional colonies).
(PDF)

S6 Fig Transcription of *cbrA* depends on CtrA. A. ChIP-Seq of the *cbrA* promoter region. In blue the plot of reads per nucleotide measured by ChIP-Seq analysis in a 600bp long region including the beginning of the coding sequence (in red). No transcriptional start site has been experimentally identified for *cbrA*. Green lines represent predicted CtrA binding site. B. Fold change in *cbrA* expression in cells after depletion of CtrA (-I, IPTG) for two hours relative to control cells expressing CtrA (+I). Expression of *cbrA* was normalized to the expression of the control gene *smc00128*. Shown are data from a representative biological replicate. Error bars indicate standard error. C. Beta-galactosidase activity assay using a LacZ fusion of the *cbrA* promoter in cells after depletion of CtrA (-I, IPTG) for two hours relative to control cells expressing CtrA (+I).
(PDF)

S7 Fig CtrA depletion in strains BM249 (Δ*ctrA* + P_lac_-*ctrA*) and EB1441 (Δ*ctrA* + P_lac_-*ctrA* + Δ*minCDE*). Bar corresponds to 2 μm. (PDF)
