# Supplementary material for: Cell Cycle Control by the Master Regulator CtrA in Sinorhizobium meliloti
Source: PLoS Genet. 2015 May 15;11(5):e1005232. doi: 10.1371/journal.pgen.1005232 (PMC4433202; doi:10.1371/journal.pgen.1005232)
Supplement: S1 Text — (PDF) [file pgen.1005232.s001.pdf]

SUPPORTING INFORMATION

Cell cycle control by the master regulator CtrA in *Sinorhizobium meliloti*

SUPPPORTING METHODS

Plasmids construction

Deletion cassettes for *ctrA* and *rcdA*, were obtained amplyfing by PCR (Pfu-turbo, Agilent) two fragments of about 1000 bp long corresponding to the upstream (primers named P1–P2, see Table S8) and downstream (primers named P3–P4, see Table S5) regions of the target genes. The fragments obtained were digested with specific restriction enzymes for directional forced cloning along with the tetracycline resistance cassette into pNPTS138 [71] (Skerker et al. 2005). All plasmids were sequenced for verification. The first six and last 12 codons of each gene deleted were left intact to protect against disruption of possible regulatory signals for adjacent genes.

For complementation plasmids, *ctrA* and *rcdA* and their putative promoter regions were amplified by PCR using the Rm1021 genomic DNA as template and primers named P1 and P4 were used for *ctrA* and *rcdA* (Table S5). Fragments were gel purified and cloned into the low-copy vector pMR10 [59] (Roberts et al. 1996).

The *ctrA* gene for depletion test *in vivo* was amplified from genomic DNA of *S. meliloti* Rm1021 by PCR using primers listed in table S5, digested by restriction (NdeI and EcoRI) and ligated in pSRKKm (previously digested with the same enzymes), generating pSRKKm-*ctrA*, which was transferred to Rm1021 by electroporation; similarly pSRKKm *ctrAΔ3A* was constructed using primers pSMc00654-P0-*NdeI*-ctrA-fw and pSMc00654Δ3A-*EcoRI*-ctrA-rev. For pSRKGm *rcdA* the coding sequence of *rcdA* was amplified using primers pSMc03989-P0-rcdA-fw and pSMc03989-P6-*KpnI*-rcdA-rev, digested with KpnI and cloned in pSRKGm sequentially digested with NdeI and KpnI, before the KpnI digestion the NdeI restriction site was restored using a DNA end repair kit (Invitrogen^{TM}). The plasmids obtained were sequenced and cloned by electroporation in Rm1021.

The *ctrADD* allele of *S. meliloti* CtrA was constructed by amplifying the predicted promoter region and coding region of *ctrA* and cloning it into a medium copy vector. A site directed mutagenesis was performed to convert the C-terminal TA residues into DD residues and transformed into *E. coli* DH5α.

For β-galactosidase assay, plasmids were constructed by directional forced cloning in a derived version of pRKlac290 [60] (Alley et al. 1991), carrying a gentamycin cassette inserted in the EcoRI site (pRKlac290Gm). Fragments (around 600 bp) of the SMc00059 *(divJ),* SMc00765 *(mcpZ),* SMc03037 *(flaA)*, SMc04114 *(pilA1),* SMc00654 *(ctrA),* SMc00021 *(ccrM)* and SMc03989 *(rcdA)* promoter regions were amplified with their respective primers in Table S5 digested with the appropriate restriction enzymes and cloned in pRKlac290Gm restricted with the same enzymes.

Real time (Fig S3)

The relative amount of the three replicons in different backgrounds was determined by real-time PCR using specific primers for each replicon rpoE1 (chromosome), minD (pSymB) and nodC (pSymA); for primers sequences see table S6. Reactions were set up as previously described [72] (Trabelsi et al.,2009). A calibration curve was generated using *S. meliloti* DNA extract, prepared from *S. meliloti* wild type cells in exponential growth (OD_{600}=0.6) or from *BM249* cells after 8 hours depletion using the DNeasy Blood & Tissue Kit (QIAgen).

SUPPLEMENTARY REFERENCES

73. Galibert F, Finan TM, Long SR, Puhler A, Abola P, et al. (2001) The composite genome of the legume symbiont Sinorhizobium meliloti. Science 293: 668-672.
71. Skerker JM, Prasol MS, Perchuk BS, Biondi EG, Laub MT (2005) Two-component signal transduction pathways regulating growth and cell cycle progression in a bacterium: a system-level analysis. PLoS Biol 3: e334.
72. Trabelsi D, Pini F, Aouani ME, Bazzicalupo M, Mengoni A (2009) Development of real-time PCR assay for detection and quantification of Sinorhizobium meliloti in soil and plant tissue. Lett Appl Microbiol 48: 355-361.
