# Supplementary material for: Cell Cycle Control by the Master Regulator CtrA in Sinorhizobium meliloti
Source: PLoS Genet. 2015 May 15;11(5):e1005232. doi: 10.1371/journal.pgen.1005232 (PMC4433202; doi:10.1371/journal.pgen.1005232)
Supplement: S1 Table — (PDF) [file pgen.1005232.s002.pdf]

Table S1 Transduction of *tetR* deletion of *ctrA* in different genetic backgrounds.

Recipient strain	Number of transduced (cfu/ml)
Empty vector	0*
ctrA C. crescentus	320
ctrA S.meliloti	310
P_{lac}-*ctrA* (no IPTG)	0*
P_{lac}-*ctrA* (IPTG 1mM)	65

*Results confirmed after 7 days
