# Supplementary material for: Cell Cycle Control by the Master Regulator CtrA in Sinorhizobium meliloti
Source: PLoS Genet. 2015 May 15;11(5):e1005232. doi: 10.1371/journal.pgen.1005232 (PMC4433202; doi:10.1371/journal.pgen.1005232)
Supplement: S4 Table — (PDF) [file pgen.1005232.s005.pdf]

Table S4 ChIP-Seq best hits.

Replicon	Peak	Upstream orf	Annotation	Downstream orf	Annotation2	Phase CC*	Microarrays
Chromosome	1867	SMc02792 op		SMc02791 ov			
Chromosome	81241	SMc02589	*16S-23S*	SMc02938			
Chromosome	81422	SMc02938 op	*16S-23S*	SMc02676			
Chromosome	85545		*16S-23S*	SMc02668 ov			
Chromosome	158487	SMc04117		ISRm22		5	yes
Chromosome	160449	SMc04115		SMc04114	*pilA1*	5	yes
Chromosome	161473	SMc04113 op		SMc04112	*cpaB1*	4	
Chromosome	172062	SMc02824		SMc02825		5	
Chromosome	197234	SMc02848		SMc02849		5	yes
Chromosome	207046	SMc02855		SMc02856			
Chromosome	285698	SMc00331 ov		SMc00435 op			
Chromosome	292793	SMc00336		SMc00337		5	
Chromosome	316400	SMc00360		SMc00361 op			
Chromosome	355909	SMc00404		Smc00405 op		6	
Chromosome	388357	SMc01163		SMc01162			
Chromosome	420462	SMc01129 op		SMc04593 op			
Chromosome	448817	SMc01107		SMc01106 op		5	
Chromosome	482999	SMc04434	*rpmH*	SMc01719	*mcpT*	3	yes
Chromosome	562831	SMc02150		SMc02149			
Chromosome	578057	SMc02135	*apaG*	SMc02134			
Chromosome	593430	SMc02229		SMc02230		4	
Chromosome	652635	SMc02278		SMc02279		6	
Chromosome	688485	SMc02316 op		SMc02317 op			
Chromosome	701834	SMc03003 op		SMc03004	*mcpE*	4	yes
Chromosome	730958	SMc03036 op	*fliP*	SMc03037	*flaA*	5	yes
Chromosome	732509	SMc03037 op	*flaA*	SMc03038	*flaB*	5	yes
Chromosome	734005	SMc03038 op	*flaB*	SMc03039	*flaD*	4	yes
Chromosome	735514	SMc03039 op	*flaD*	SMc03040	*flaC*	4	yes
Chromosome	742209	SMc03045 op		SMc03046		4	
Chromosome	754612	SMc03058		SMc03059	*folD2*		
Chromosome	775481	SMc00764 op		SMc00765	*mcpZ*	5	yes
Chromosome	816882	SMc00795		SMc00796			
Chromosome	859521	SMc00924		SMc00923		5	yes
Chromosome	894948	SMc00932	*mutL*	SMc00888		5	yes
Chromosome	914813	SMc00873 op		SMc00928		5	
Chromosome	938623	SMc00849		SMc00993 op			
Chromosome	943503	SMc00991		SMc00990 op		5	yes
Chromosome	948684	SMc00987		SMc00986		4	yes
Chromosome	954495	SMc00983		SMc00982		5	yes
Chromosome	958673	SMc00980		SMc00979 ov			
Chromosome	963369	SMc00976 op		SMc00975	*mcpU*	na	yes
Chromosome	984405	SMc00069	*pdxH*	SMc00002			
Chromosome	1014348	SMc00080		SMc00021	*ccrM*	5	
Chromosome	1045597	SMc00032 op		SMc00033		2	yes
Chromosome	1053531	SMc00097	*gst2*	SMc00038			
Chromosome	1093187	SMc00456		SMc00059	*divJ*	5	yes
Chromosome	1097769	SMc00120		SMc00062		4	
Chromosome	1099135	SMc00063 op		SMc00128 op			
Chromosome	1121434	SMc02369	*pleC*	SMc02370 op		4	
Chromosome	1144812	SMc02391		SMc02392		5	yes
Chromosome	1148290	SMc02461		SMc02396			
Chromosome	1180972	SMc02641	*rkpK*	SMc02640 op			
Chromosome	1206591	SMc02556		SMc02555 op			
Chromosome	1216617	SMc02544		SMc02543			
Chromosome	1253975	SMc00583 ov					

Chromosome	1255985	SMc00584		SMc00585	*pepA*		
Chromosome	1272236	SMc00601	*pgsA*	SMc00602 ov			
Chromosome	1275112	SMc00603		SMc00604	*ropB1*	5	
Chromosome	1314272	SMc00604 op		SMc00605 op			
Chromosome	1315214	SMc01768 op		SMc01769		5	
Chromosome	1339497	SMc01792		SMc01793 op		6	
Chromosome	1341260	SMc01794		SMc01795		4	
Chromosome	1407669	SMc01374		SMc01373 op			
Chromosome	1411567	SMc01370 op		SMc01369 op			
Chromosome	1426674	SMc01358		SMc01357		5	
Chromosome	1445018	SMc01336	*rne*	SMc01335	*amiC*		
Chromosome	1473673	SMc01315		SMc01314	*rpsL*	6	
Chromosome	1518749	SMc01257		SMc01256	*sda*		
Chromosome	1523666	SMc00996		SMc00997 op		5	yes
Chromosome	1524724	SMc00997		SMc00998		5	yes
Chromosome	1526931	SMc00999		SMc01000 op		5	yes
Chromosome	1527648	SMc01000		SMc01001			
Chromosome	1531152	SMc01003		SMc01004 op			
Chromosome	1539802	SMc01014		SMc01015		4	
Chromosome	1555331	SMc04486		SMc01029		5	
Chromosome	1614071	SMc02095 op		SMc02094	*omp*		
Chromosome	1644082	SMc02073 over		SMc02072	*argS*		
Chromosome	1655838	SMc02062 op		SMc02061	*bioS*	4	
Chromosome	1656896	SMc04539 op		SMc02060	*lppB*	5	yes
Chromosome	1666957	SMc02051		SMc04537 op			
Chromosome	1677049	SMc01241		SMc01240	*TRm5*		
Chromosome	1728294	SMc01202		SMc01200 op			
Chromosome	1743943	SMc01187	*rlpA*	SMc01186		5	
Chromosome	1850507	SMc00280 op		SMc00281 op			
Chromosome	1859585	SMc00289	*cspA5*	SMc00290	*gloA*		
Chromosome	1890119	SMc00540 op		SMc00539		5	
Chromosome	1905408	SMc00508 op		SMc00507		6	
Chromosome	1960225	SMc00466 op		SMc00548		5	
Chromosome	2008541	SMc04549 op		SMc00159		5	
Chromosome	2019845	SMc00150		SMc00149	*fumC*		
Chromosome	2033247	SMc00138 op		SMc00137			
Chromosome	2040004	SMc04211		SMc04212		5	
Chromosome	2051187	SMc04225		SMc04226 op		5	
Chromosome	2053988	SMc04227	*mcpV*	SMc04228		5	
Chromosome	2129268	SMc04310 op		SMc04311			
Chromosome	2206120	SMc04280		SMc04283 op			
Chromosome	2236639	SMc04352		SMc04357			
Chromosome	2276741	SMc01431	*ilvI*	SMc01432		5	yes
Chromosome	2310691	SMc01463		SMc01464			
Chromosome	2318021	SMc01469	*mcpW*	SMc01470		5	yes
Chromosome	2338689	SMc01872		SMc01871 over		5	
Chromosome	2339449	SMc01871	*ddl*	SMc01870 op		5	
Chromosome	2356519	SMc01857	*mraZ*	SMc05022			
Chromosome	2367946	SMc01847 op		SMc01846		6	
Chromosome	2382359	SMc01574		SMc01575			
Chromosome	2386066	SMc01579		SMc01582		5	yes
Chromosome	2455001	SMc01656 op		SMc01657	*foxA*		
Chromosome	2490684	SMc01550 op		SMc01551			
Chromosome	2500983	SMc01561		SMc01562 op		5	yes
Chromosome	2504020	SMc01563	*rpoD*	SMc01564 op			
Chromosome	2521220	SMc01585 op		SMc01586		1	
Chromosome	2529319	SMc02678		SMc03744			
Chromosome	2829868	SMc02447		SMc02448 op		5	

Chromosome	2838047	SMc00744		SMc00743		5	yes
Chromosome	2842636	SMc00741		SMc00740		6	
Chromosome	2863347	SMc00718		SMc00717		5	
Chromosome	2888384	SMc00690 op		SMc00689			
Chromosome	2920148	SMc00658		SMc00657	*sciP*	5	yes
Chromosome	2921700	SMc00655		SMc00654	*ctrA*	5	yes
Chromosome	2923528	SMc00653	*cheY like*	SMc00652 op		5	
Chromosome	2924524	SMc00652	*chpT*	SMc00651		5	
Chromosome	2939040	SMc00639		SMc00638 op		4	
Chromosome	2940068	SMc00638		SMc00637 op		4	yes
Chromosome	2947317	SMc02942	*pal*	SMc04461 op			
Chromosome	2951381	SMc03958	*tolQ*	SMc03959			
Chromosome	2984340	SMc03985	*cyaF2*	SMc03986 op			
Chromosome	2986298	SMc03988 ov		SMc03989	*rcdA*	na	
Chromosome	3005541	SMc04010		SMc04011 op			
Chromosome	3007290	SMc04011	*tacA*	SMc04012	*pepF*	3	yes
Chromosome	3019439	SMc04021 ov		SMc04022		5	
Chromosome	3021963	SMc04023	*exoN2*	SMc04024		5	
Chromosome	3047800	SMc04043		SMc04044	*cpdR1*	6	
Chromosome	3063682	SMc04058 op		SMc04059		5	yes
Chromosome	3136864	SMc03174		SMc03175		5	yes
Chromosome	3140797	SMc03177 ov		SMc03178		5	
Chromosome	3177609	SMc03150		SMc03149			
Chromosome	3185520	SMc03145 op		SMc03144			
Chromosome	3187871	SMc03143		SMc03142		1	yes
Chromosome	3214858	SMc03224		SMc03122 op			
Chromosome	3246512	SMc03098 op		SMc03097			
Chromosome	3306691	SMc02489 op		SMc02488			
Chromosome	3334452	SMc02463	*sdhC*	SMc03225			
Chromosome	3349661	SMc03241		SMc03242 op			
Chromosome	3400012	SMc03284 op		SMc03285 op			
Chromosome	3473046	SMc05025 ov		SMc03808	*ftsK*	5	
Chromosome	3499665	SMc03832		SMc03833 op			
Chromosome	3501760	SMc03835	*ligT*	SMc03836 op			
Chromosome	3631610	SMc04089		SMc04090 op			
Chromosome	3645959	SMc02801 ov	*parB*	SMc02800 op	*parA*		
Chromosome	3646109	SMc02801	*parB*	SMc02800 ov			
Chromosome	3650640	SMc02797 ov		SMc02796 op			
pSymA	199154	SMa0372 ov		/			
pSymA	238077	ISRm1		/			
pSymA	420636	SMa0773 ov	*noeA*	SMa0774	*noeB*		
pSymA	441538	SMa0805 op	*gabD4*	SMa0806	*syrB3*		
pSymA	547164	SMa0983 ov		/			
pSymA	551279	ISRm5		/			
pSymA	564988	SMa1016		SMa1017			
pSymA	743233	SMa1355		ISRm3			
pSymA	786643	SMa1427		SMa1428			
pSymA	869098	SMa1570	*pilA2*	SMa1572 op			
pSymA	876501	SMa1582 ov		SMa1583 op			
pSymA	880354	SMa1586	*syrB2*	SMa1587	*eglC*		
pSymA	893864	SMa1600 op		SMa1602 op			
pSymA	963113	SMa1706 ov		SMa1711	*argI2*	6	
pSymA	1262738	SMa2251 op		SMa2253			
pSymA	1328619	SMa2355 op	*DNA pol IV*				
pSymA	1353544	SMa2395	*repA2*	SMa0002	*fdoG*	3	
pSymB	54856	SM_b20043 op		SM_b20044 op	*repC1*	3	
pSymB	70369	SM_b20059 ov	*ISRm5*	/			
pSymB	71554	ISRm5		SM_b22003 op			

pSymB	133432	SM_b20121 ov		SM_b20122 op			
pSymB	231498	SM_b22001 op		SM_b20225 op			
pSymB	311741	ISRm2011		/			
pSymB	369437	SM_b20358 op		SM_b20359			
pSymB	470306	SM_b20457		SM_b20458			
pSymB	636455	SM_b20843	*algI*	SM_b21013 op			
pSymB	733767	SM_b21090 op		SM_b21091	*lysM*	5	yes
pSymB	800766	ISRm1		SM_b21235 op			
pSymB	813166	SM_b21244		SM_b21245	*exoF3*		
pSymB	1174129	SM_b20946	*exoY*	SM_b20947	*exoX*	6	
pSymB	1237946	SM_b21001 ov		SM_b21002 op			
pSymB	1249096	SM_b20846 op		SM_b21676 op			
pSymB	1332437	SM_b21401 op		SM_b21402			
pSymB	1402899	SM_b21478 op		SM_b21479		5	
pSymB	1427713	SM_b21502		SM_b21503 op		5	yes
pSymB	1432972	SM_b21506		SM_b21507		5	yes
pSymB	1438191	SM_b21513	*wzx2*	SM_b21514	*hemK2*	5	yes
pSymB	1447964	SM_b21524 over	*minCDE*	SM_b20647 op		4	yes
pSymB	1455434	SM_b20652	*asnB*	SM_b20654			
pSymB	1460822	SM_b20659		SMb20660 op			
pSymB	1466911	ISRm17		/			
pSymB	1528966	SM_b20723 ov		SM_b20724			
pSymB	1551923	SM_b20758		SM_b20759	*phnG*	2	
pSymB	1557320	SM_b20765 op		ISRm22		2	
pSymB	1557790	ISRm22		/			
pSymB	1558328	ISRm22		/			
pSymB	1610618	SM_b20596		SM_b20597		3	

Green represents genes possibly controlled by CtrA (CDS not overlapping with the binding site). Red color represents opposite frame or overlapping binding sites with the CDS.
