# Supplementary material for: Cell Cycle Control by the Master Regulator CtrA in Sinorhizobium meliloti
Source: PLoS Genet. 2015 May 15;11(5):e1005232. doi: 10.1371/journal.pgen.1005232 (PMC4433202; doi:10.1371/journal.pgen.1005232)
Supplement: S5 Table — (PDF) [file pgen.1005232.s006.pdf]

Table S5 Transduction of *tetR* deletion of *rcdA* in different genetic backgrounds.

**Recipient strain**	**Number of transduced (cfu/ml)**
Empty vector	0
rcdA S.meliloti	54
P_{lac}-	0
P_{lac}-*rcdA* (no IPTG)	0
P_{lac}-*rcdA* (IPTG 80μM)	200

* Colonies counted after 6 days
