# Supplementary material for: Cell Cycle Control by the Master Regulator CtrA in Sinorhizobium meliloti
Source: PLoS Genet. 2015 May 15;11(5):e1005232. doi: 10.1371/journal.pgen.1005232 (PMC4433202; doi:10.1371/journal.pgen.1005232)
Supplement: S6 Table — (PDF) [file pgen.1005232.s007.pdf]

Table S6 Strains and plasmids used in this work.

Organism or plasmid	Strain or plasmid name	Description	Resistance	Source
S. meliloti	Rm1021	SU47 *str-21*	Sm	[73]
	BM130	Rm1021 + pMR10 *ctrA*(C.cre)	Sm, Km	This work
	BM132	Rm1021 + pMR10 *ctrA*(S.mel)	Sm, Km	This work
	BM240	Rm1021 + pSRKKm *ctrA*(S.mel)	Sm, Km	[33]
	BM146	Rm1021ΔctrA + pMR10 *ctrA*(C.cre)	Sm, Km, Tc	This work
	BM196	Rm1021ΔctrA + pMR10 *ctrA*(S.mel)	Sm, Km, Tc	This work
	BM249	Rm1021Δ*ctrA* + pSRKKm *ctrA*(S.mel)	Sm, Km, Tc 1mM IPTG	This work
	EB776	Rm1021 + pSRKKm + *ctrA* Δ3A (S. mel)	Sm, Km	This work
	BM587	Rm1021 + pMR10 + *rcdA*	Sm, Km	This work
	EB1024	Rm1021 + pSRKGm *rcdA* (S. mel)	Sm, Gm	This work
	EB882	Rm1021 Δ*rcdA* + pMR10 *rcdA* (S. mel)	Sm, Km, Tc	This work
	EB1202	Rm1021 Δ*rcdA* + pMR10	Sm, Km, Tc	This work
	EB1439	Rm1021 Δ*minCDE* (SmK850)	Sm, Spec	[37]
	EB1441	Rm1021 BM249 Δ*minCDE*	Sm, Spec, Tet, Kan	This work
	EB1051	Rm1021 Δ*rcdA* + pSRKGm *rcdA* (S. mel)	Sm, Gm, Tc, 80 µM IPTG	This work
		Rm1021 Δ*cpdR1uidA*	Sm, Sp	[29]
	EB1108	Rm1021 + pRKlac290Gm P*flaA* (S. mel)	Sm, Gm, Tc	This work
	EB1084	Rm1021 + pRKlac290Gm P*mcpZ* (S. mel)	Sm, Gm, Tc	This work
	EB1155	Rm1021 + pRKlac290Gm P*pilA1* (S. mel)	Sm, Gm, Tc	This work
	EB1334	Rm1021 + pRKlac290Gm P*divJ* (S. mel)	Sm, Gm, Tc	This work
	EB1106	Rm1021 + pRKlac290Gm + P1ctrA (Sm)	Sm, Gm, Tc	This work
	EB1154	Rm1021 + pRKlac290Gm + P2ctrA (Sm)	Sm, Gm, Tc	This work
	EB1331	Rm1021 + pRKlac290Gm + P1P2ctrA (Sm)	Sm, Gm, Tc	This work
	EB1115	Rm1021Δ*ctrA* + pSRKKm *ctrA*(S.mel) + pRKlac290Gm P*flaA* (S. mel)	Sm, Gm, Km, Tc, IPTG 1mM	This work
	EB1085	Rm1021Δ*ctrA* + pSRKKm *ctrA*(S.mel) + pRKlac290Gm P*mcpZ* (S. mel)	Sm, Gm, Km, Tc, IPTG 1mM	This work
	EB1157	Rm1021Δ*ctrA* + pSRKKm *ctrA*(S.mel) + pRKlac290Gm P*pilA1* (S. mel)	Sm, Gm, Km, Tc, IPTG 1mM	This work
	EB1333	Rm1021Δ*ctrA* + pSRKKm *ctrA*(S.mel) + pRKlac290Gm P*divJ* (S. mel)	Sm, Gm, Km, Tc, IPTG 1mM	This work
	EB1101	Rm1021Δ*ctrA* + pSRKKm *ctrA*(S.mel) + pRKlac290Gm P1*ctrA* (S. mel)	Sm, Gm, Km, Tc, IPTG 1mM	This work
	EB1156	Rm1021Δ*ctrA* + pSRKKm *ctrA*(S.mel) + pRKlac290Gm P2*ctrA* (S. mel)	Sm, Gm, Km, Tc, IPTG 1mM	This work
	EB1336	Rm1021Δ*ctrA* + pSRKKm *ctrA*(S.mel) + pRKlac290Gm P1P2*ctrA* (S. mel)	Sm, Gm, Km, Tc, IPTG 1mM	This work
E. coli	S17-1	*recA*, *pro*, *hsdR*, *RP4-2-Tc::Mu-km::Tn7*	-	(Simon et al. 1983)
Bacteriophage	ΦM12	Transducing phage		[62]
Plasmids				

General purpose vectors	pNTPS138	Suicide vector, *oriT*, *sacB*	Km	D. Alley
	pMR10	Broad host-range cloning vector, low copy number	Km	(Roberts et al. 1996)
	pSRKKm	pBBR1MCS-2-derived broad-host-range expression vector containing *lac* promoter and *lacI*q, *lacZ+*	Km	[36]
	pSRKGm	pBBR1MCS-5-derived broad-host-range expression vector containing *lac* promoter and *lacI*q, *lacZ+*	Gm	[36]
	pRKlac290			[60]
	pRKlac290Gm	pRKlac290 reporter vector with a Gentamycin cassette between EcoRI site	Gm, Tc	This work
	pBBR1MCS5		Gm	[73]
Deletion plasmids	pΔ*ctrA*	pNPTS138-Tc deletion cassette for *ctrA*	Km, Tc	This work
	pΔ*rcdA*	pNPTS138-Tc deletion cassette for *rcdA*	Km, Tc	This work
Gene replacement plasmid	p*ctrADD*			This work
Complementation plasmids	pMR10*ctrA*	pMR10 containing the *ctrA* locus	Km	This work
	pMR10*rcdA*	pMR10 containing the *rcdA* locus	Km	This work
pBBR1MCS5 derivatives	pBBR1MCS5*ctrA*DD	pBBR1MCS5-p*ctrA* (S.mel) *ctrA*DD (S.mel)	Gm	This work
	pBBR1MCS5 *ctrA*∆3A	pBBR1MCS5-p*ctrA* (S.mel) *ctrA* ∆3A (S.mel)		This work
Overexpression/depletion plasmids	pSRKKm*ctrA*	pSRKKm containing *ctrA* inserted between NdeI and KpnI sites	Km	(Pini et al. 2013)
	pSRKKm*ctrA Δ3A*	pSRKKm containing *ctrA Δ3A* inserted between NdeI and KpnI sites	Km	This work
	pSRKGm*rcdA*	pSRKGm containing *rcdA* inserted between NdeI and KpnI sites	Gm	This work
Reporter plasmids	pRKlac290Gm-PSmc00059	*SMc00059 (divJ)* promoter–*lacZ* fusion in pRKlac290Gm	Gm, Tc	This work
	pRKlac290Gm-PSmc00765	*SMc00765 (mcpZ)* promoter–*lacZ* fusion in pRKlac290Gm	Gm, Tc	This work
	pRKlac290Gm-PSmc03037	*SMc03037 (flaA)* promoter–*lacZ* fusion in pRKlac290Gm	Gm, Tc	This work
	pRKlac290Gm-PSmc04114	*SMc04114 (pilA1)* promoter–*lacZ* fusion in pRKlac290Gm	Gm, Tc	This work
	pRKlac290Gm-PSmc03989	*SMc03989 (rcdA)* promoter–*lacZ* fusion in pRKlac290Gm	Gm, Tc	This work
	pRKlac290Gm-PSmc00021	*SMc00021 (ccrM)* promoter–*lacZ* fusion in pRKlac290Gm	Gm, Tc	This work
	pRKlac290Gm-P1Smc00654	*SMc00654 (ctrA)* promoterP1–*lacZ* fusion in pRKlac290Gm	Gm, Tc	This work
	pRKlac290Gm-P2Smc00654	*SMc00654 (ctrA)* promoterP2–*lacZ* fusion in pRKlac290Gm	Gm, Tc	This work
	pRKlac290Gm-P1P2Smc00654	*SMc00654 (ctrA)* promoterP1P2–*lacZ* fusion in pRKlac290Gm	Gm, Tc	This work
