# Supplementary material for: Cell Cycle Control by the Master Regulator CtrA in Sinorhizobium meliloti
Source: PLoS Genet. 2015 May 15;11(5):e1005232. doi: 10.1371/journal.pgen.1005232 (PMC4433202; doi:10.1371/journal.pgen.1005232)
Supplement: S7 Table — (PDF) [file pgen.1005232.s008.pdf]

Table S7 Primers (5'-3') used in this work.

pSMc00654-*HindIII*-P1	GGAAGCTTCACAATGCGCCGATTCAACA	This work
pSMc00654-*EcoRI*-P2	GGGAATTCGATCAGTAGAACCCGCAT	This work
pSMc00654-*EcoRI*-P3	GGGAATTCGAGCCGGAAGGCAGCGAC	This work
pSMc00654-*SphI*-P4	GGGCATGCTTGCCGAGGCTGCGGAATAG	This work
pSMc00654-ctrA-seq-fw	TTGGCCCTTCACGCGATCGA	This work
pSMc00654-ctrA-seq-rev	ATGGCGATCGACGTCGTATC	This work
pSMc00654-P0-*NdeI*-ctrA-fw	GGCATATGCGGGTTCTACTGATCGAAG	This work
pSMc00654Δ3-*EcoRI*-ctrA-rev	GAATTCACAGGTAGTCGCTGCCTT	This work
pSMc02139-P1-(HindIII)	AAGCTTTGATGACGTGGAACTTCT	This work
pSMc02139-P2-(EcoRI)	GGGAATTCGTCAGTCCAGTTCATCTCTG	This work
pSMc02139-P3-(EcoRI)	GGGAATTCGCCGAGCGCCGGCGCATG	This work
pSMc02139-P4-(BamHI)	GGATCCCCGTGCGCGGCGGCATGA	This work
pSMc02139-P0-*NdeI*-gcrA-fw	CATATGAACGGACTGACGAGCGG	This work
pSMc02139-P6-*KpnI*-gcrA-rev	GGTACCGGAATGAGCAAGGCGCCTC	This work
pSMc02139-gcrA-ext-fw	CCGAAGGGGACCTGATAGA	This work
pSMc02139-gcrA-ext-rv	ATGACGTCGACATTGGGTTC	This work
pSMc03989-P1-(HindIII)	AAGCTTGCCTTATGGCCTCTTCAT	This work
pSMc03989-P2-(EcoRI)	GAATTCCAATCCTCTTTCGGACAT	This work
pSMc03989-P3-(EcoRI)	GAATTCTTTCTGGCGCGGGCGTGA	This work
pSMc03989-P4-(BamHI)	GGATCCCGCGGTAACGCGAGAGATAAGG	This work
pSMc03989-ext-fw	GTCGGACAGCACAGTTGC	This work
pSMc03989-ext-rv	AGACGTCGCGCCTGAGAT	This work
pSMc03989-P0-rcdA-fw	TCCGAAAGAGGATTGAATACC	This work
pSMc03989-P6-*KpnI*-rcdA-rev	GGGGTACCGTCATCTTCGGGCTTGACC	This work
M13Fw	GTAAAACGACGGCCAG	This work
M13Rv	CAGGAAACAGCTATGAC	This work
pPpilA1Sm-BamHI-fw	GGATCCTGTTGCCCTCGTCGATCGATACC	This work
pPpilA1Sm-XbaI-rev	TCTAGAGTCACGCAATAAATTCACGCCTTGCC	This work
pPdivJSm-BamHI-fw	GGATCCCTGCATTTTAGCGATCGTCG	This work
pPdivJSm-XbaI-rev	TCTAGACCACGAGGGATGTCCATCTGCCAGC	This work
pPflaASm-KpnI-fw	GGTACCTCGACATCGCCCGGGAGAAGG	This work
pPflaASm-XbaI-rev	TCTAGACGCGGCCATTGCGGAGTTGT	This work
pPmcpZSm-BamHI-fw	GGATCCGGCAGACCGGTCTTTGCCGG	This work
pPmcpZSm-XbaI-rev	TCTAGAGGAAAACACCTTGGCTACAGCGCGC	This work
pPrcdASm-BamHI-fw	GGATCCGAGATCGAATGAGCCTGTAT	This work
pPrcdASm-XbaI-rv	TCTAGAGCCTGTATTCCCTGGTTCCAACG	This work
pPccrMSm-BamHI-fw	GGATCCCGATCAGGCTGTCCAGCATG	This work
pPccrMSm-XbaI-rv	TCTAGACACGGGAGATTTCGGCAAGC	This work
pPctrASm-XbaI-rv	TCTAGAAGTCTTCCCCTTTTCCGCCG	This work
pP1ctrASm-XbaI-rv	TCTAGACGATACATGAGGGAATGCCG	This work
pP2ctrASm-KpnI-fw	GGTACCATGTATCGCATGAGGAGC	This work
pP1ctrASm-BamHI-fw	GGATCCCCTCTGGCGGGCAGCCTTGG	This work
pP1P2ctrASm-KpnI-fw	GGTACCCCTCTGGCGGGCAGCCTTGG	This work
rpoE1-fw	CGAGGAAGAGGTCCTGGAAT	[4]
rpoE1-rv	GACGCAGTCCTGCAACAGAT	[4]
nodC-fw	GCCGCTATCTCAATCTACGC	[4]
nodC-rv	TTGAAGCTGGGGACGATAAC	[4]
minD fw	GATTGGAAGCCTTGAGAACG	This work
minD rv	GACATGCTCAAGGTCGATGA	This work
