# Supplementary material for: Cell Cycle Control by the Master Regulator CtrA in Sinorhizobium meliloti
Source: PLoS Genet. 2015 May 15;11(5):e1005232. doi: 10.1371/journal.pgen.1005232 (PMC4433202; doi:10.1371/journal.pgen.1005232)
Supplement: S8 Table — (PDF) [file pgen.1005232.s009.pdf]

Table S8 TTSs mapping in the 54 direct targets of CtrA.

Orf code	Name	Distance upstream of TSS1$	Presence of CtrA-BS	Presence of half CtrA-BS
SM_b20652	*asnB*	21	-	+
SM_b21091	*lysM*	/	-	-
SM_b21502		/	-	-
SM_b21503		/	-	-
SM_b21506		/	+	+
SM_b21513	*wzx2*	60	+	+
SM_b21514	*hemK2*	/	+	+
SM_b21524	*minCDE*	/	+	+
SMa1016		182	+	+
SMc00033		109	-	-
SMc00038		48	-	+
SMc00059	*divJ*	/	+	+
SMc00360		180	+	+
SMc00456		/	+	+
SMc00638		/	+	+
SMc00654	*ctrA*	89	+	+
SMc00657	*sciP*	121	-	+
SMc00743		73	+	+
SMc00765	*mcpZ*	146	-	+
SMc00849		26	-	-
SMc00888		65	+	+
SMc00924		5	-	+
SMc00975	*mcpU*	49	-	+
SMc00983		/	-	+
SMc00986		32	-	+
SMc00991		96	-	+
SMc00996		/	-	+
SMc00998		16	+	+
SMc00999		7	-	+
SMc01357		49	+	+
SMc01432		43	-	+
SMc01464		10	-	+
SMc01469	*mcpW*	/	-	+
SMc01561		/	-	+
SMc01579		/	+	+
SMc01719	*mcpT*	61	-	+
SMc01792		/	-	-
SMc02060	*lppB*	81	+	+
SMc02392		166	+	+
SMc02848		6	+	+
SMc03004	*mcpE*	72	-	+
SMc03037	*flaA*	198	+	+
SMc03038	*flaB*	186	-	+
SMc03040	*flaC*	121	+	+
SMc03142		1	+	+

SMc03143		404	+	+
SMc03174		/	-	+
SMc03178		66	-	+
SMc04011	*tacA*	/	-	+
SMc04059		60	-	-
SMc04114	*pilA1*	62	+	+
SMc04115		101	+	+
SMc04117		/	-	+
SMc04280		/	-	+

/ = TSS Not identified previously (Schluter et al., 2013) or upstream the ChIP-seq peak.
In green genes represented in figure 3B.
$ = Position of TSSs with respect to the ChIp-seq peak.
+ = Presence of CtrA full or Half binding site consensus (Laub et al., 2002)
- = Absence of CtrA full or Half binding site consensus
