# Supplementary figures and images for: Cell Cycle Control by the Master Regulator CtrA in Sinorhizobium meliloti
Source: PLoS Genet. 2015 May 15;11(5):e1005232. doi: 10.1371/journal.pgen.1005232 (PMC4433202; doi:10.1371/journal.pgen.1005232)

FIGURE S1

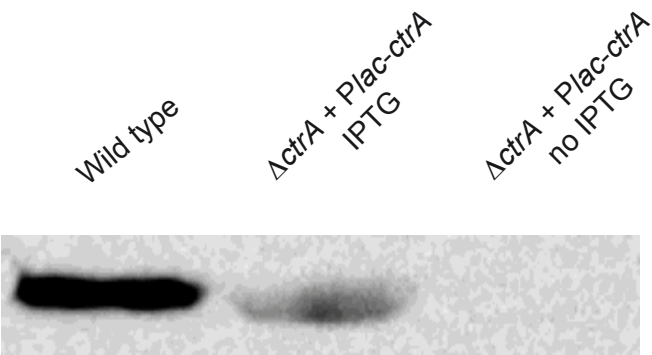

Supplement: S1 Fig — (PDF) [file pgen.1005232.s010.pdf]

FIGURE S2

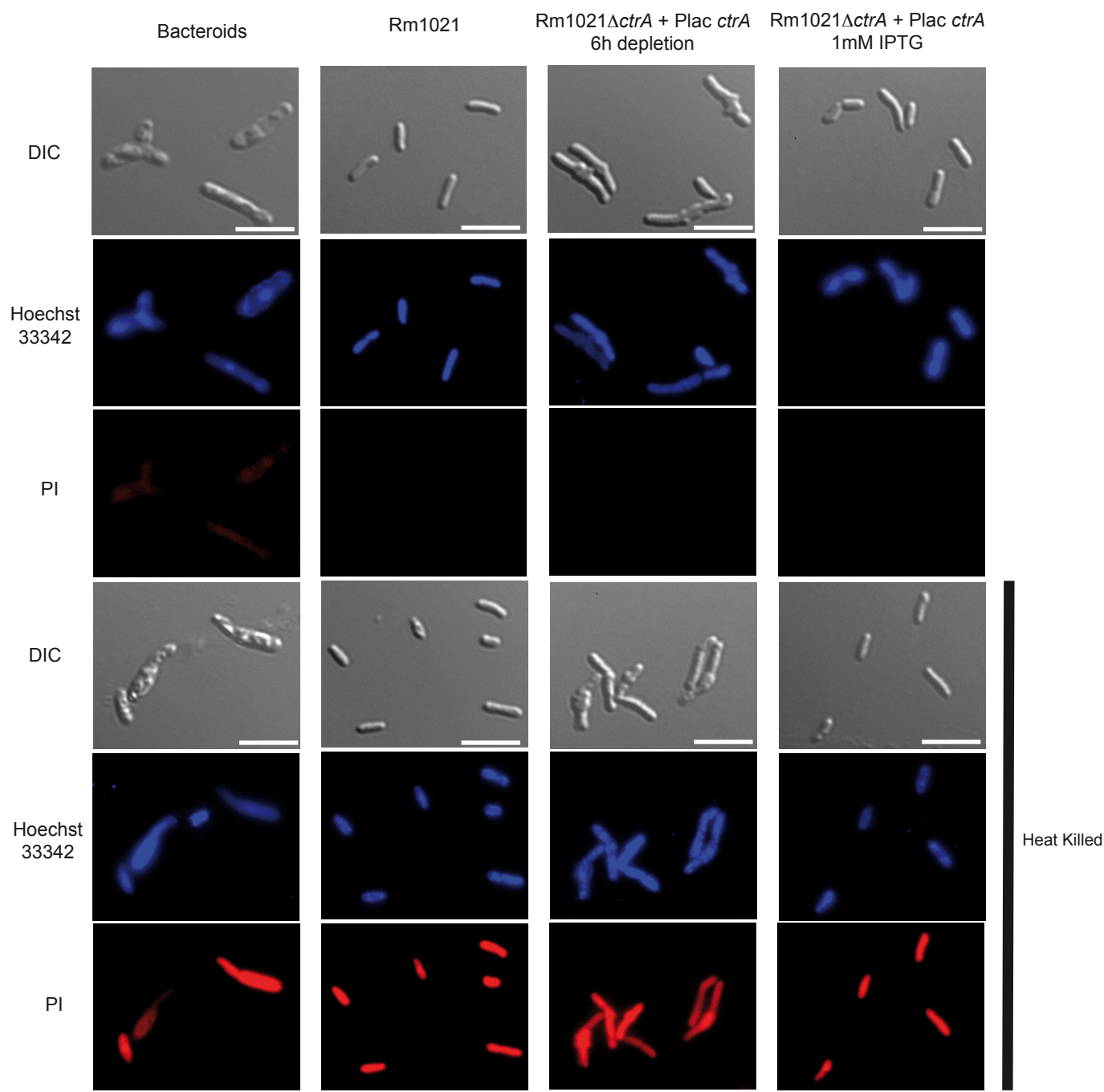

Supplement: S2 Fig — “Heat-treated” indicates 10-min treatment at 70°C, as in Mergaert et al., 2006 (Scale bar = 5 μm). (PDF) [file pgen.1005232.s011.pdf]

FIGURE S3

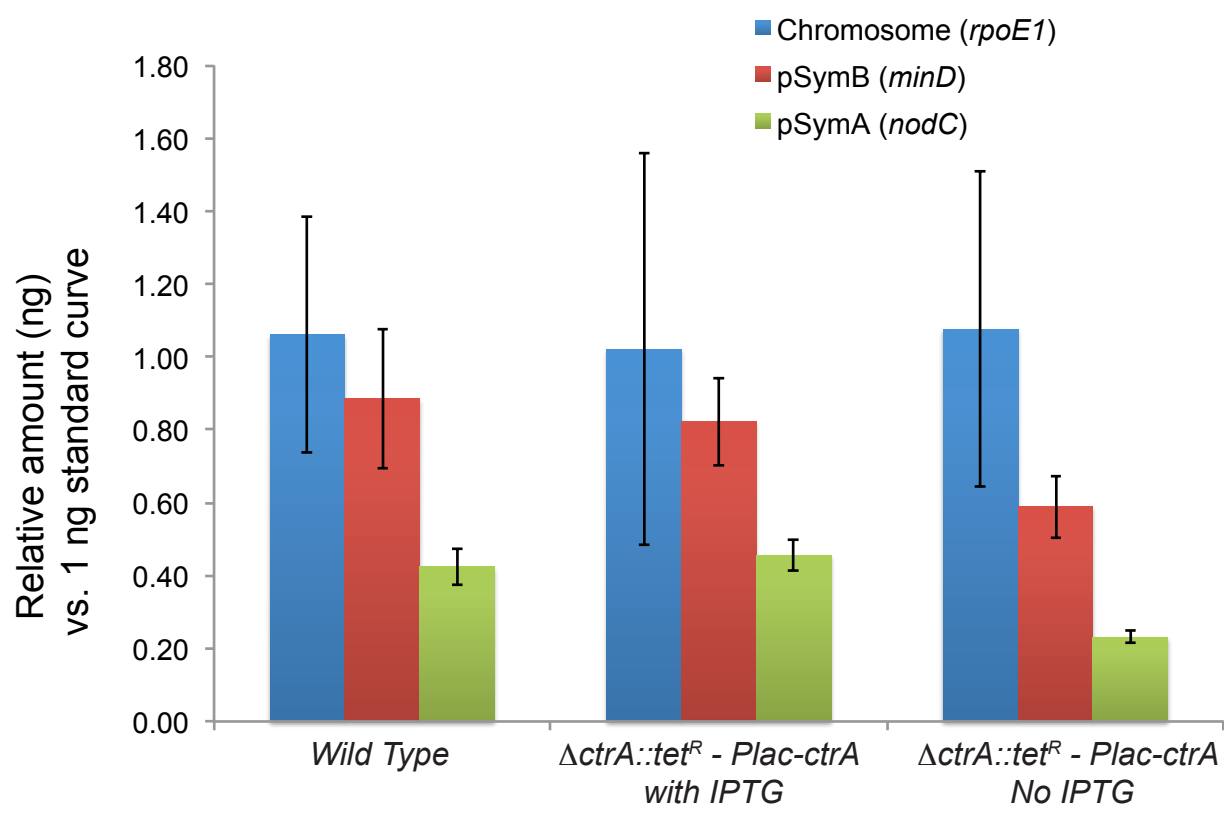

Supplement: S3 Fig — (PDF) [file pgen.1005232.s012.pdf]

Figure S4

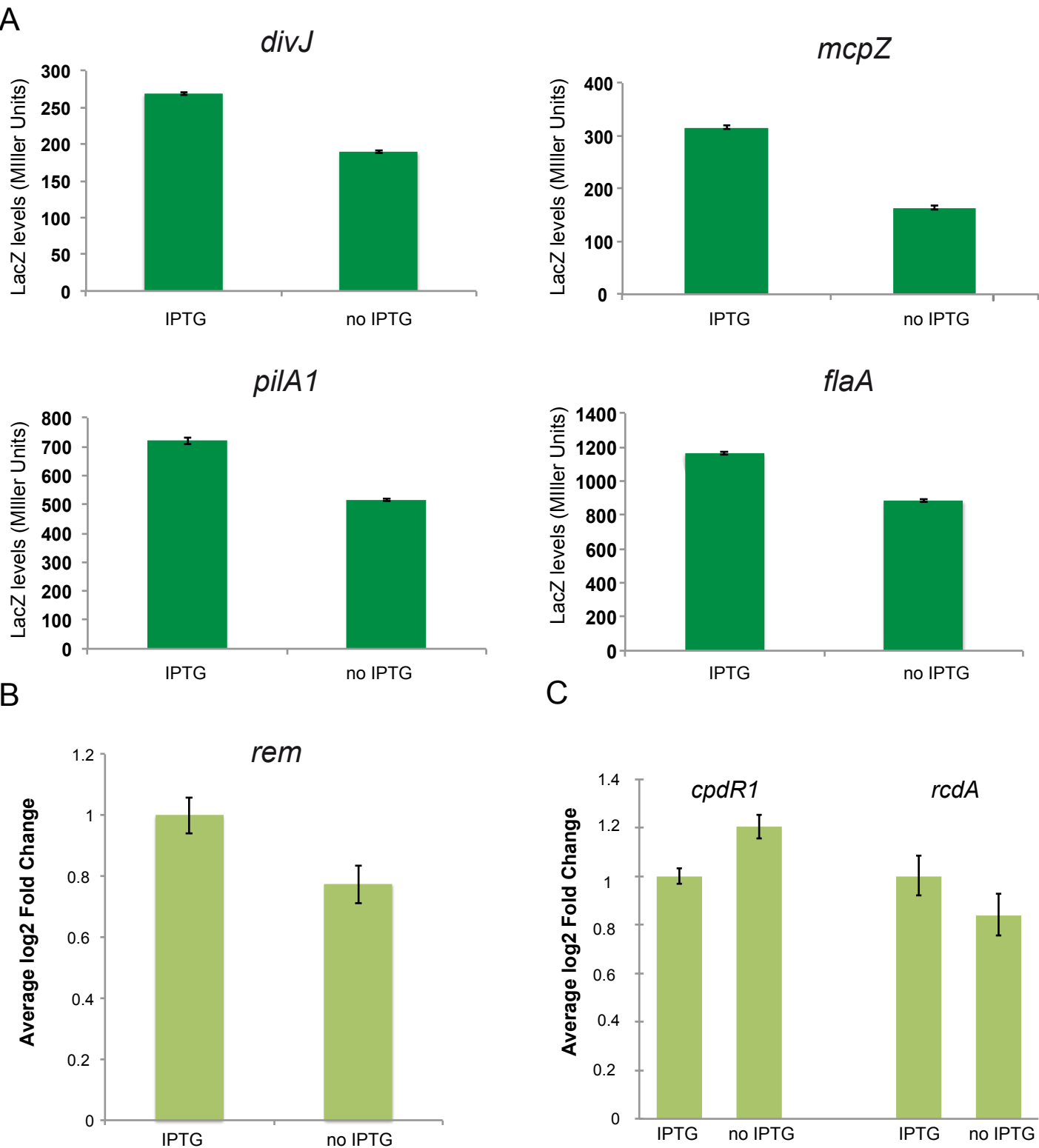

Supplement: S4 Fig — A. β-galactosidase assays of genes of Fig 3B B. Fold change in rem expression in cells after depletion of CtrA (-I, IPTG) for two hours relative to control cells expressing CtrA (+I). Expression of rem was normalized to the expression of the control gene smc00128. Shown are data from a representative biological replicate. Error bars indicate standard error. C. Fold change in cpdR1 and rcdA expression in cells after depletion of CtrA (-I, IPTG) for four hours relative to control cells expressing CtrA (+I). Expression of cpdR1 and rcdA was normalized to the expression of the control gene smc00128. Data are shown from a representative biological replicate. Error bars indicate standard error. (PDF) [file pgen.1005232.s013.pdf]

Figure S5

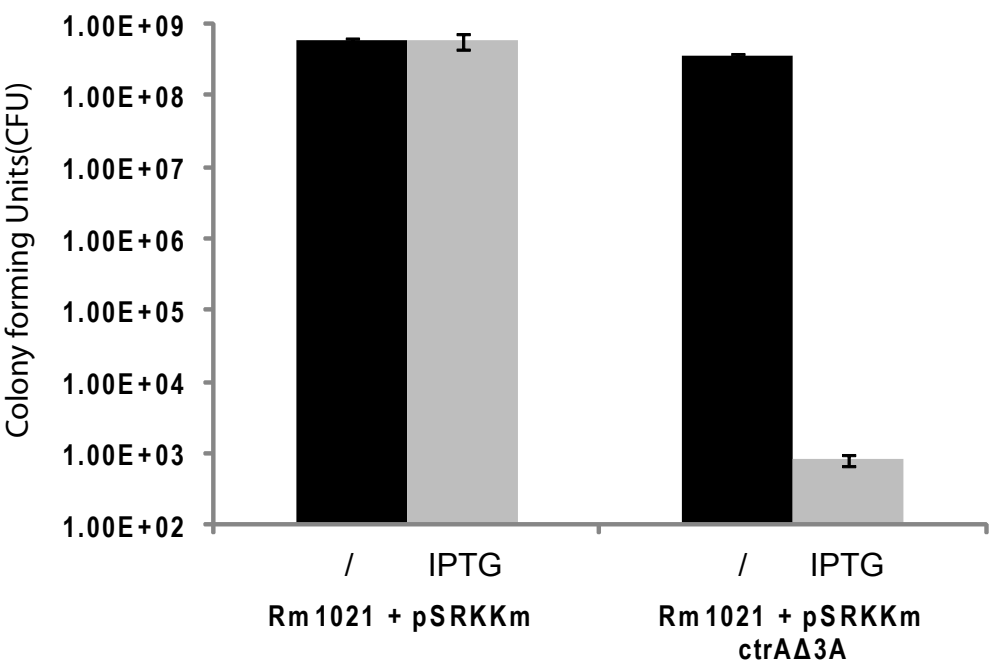

Supplement: S5 Fig — The number of colonies was determined after 4 to 5 days of growth without IPTG at 30°C (further incubation of the plates did not result in the appearance of additional colonies). (PDF) [file pgen.1005232.s014.pdf]

Figure S6

A

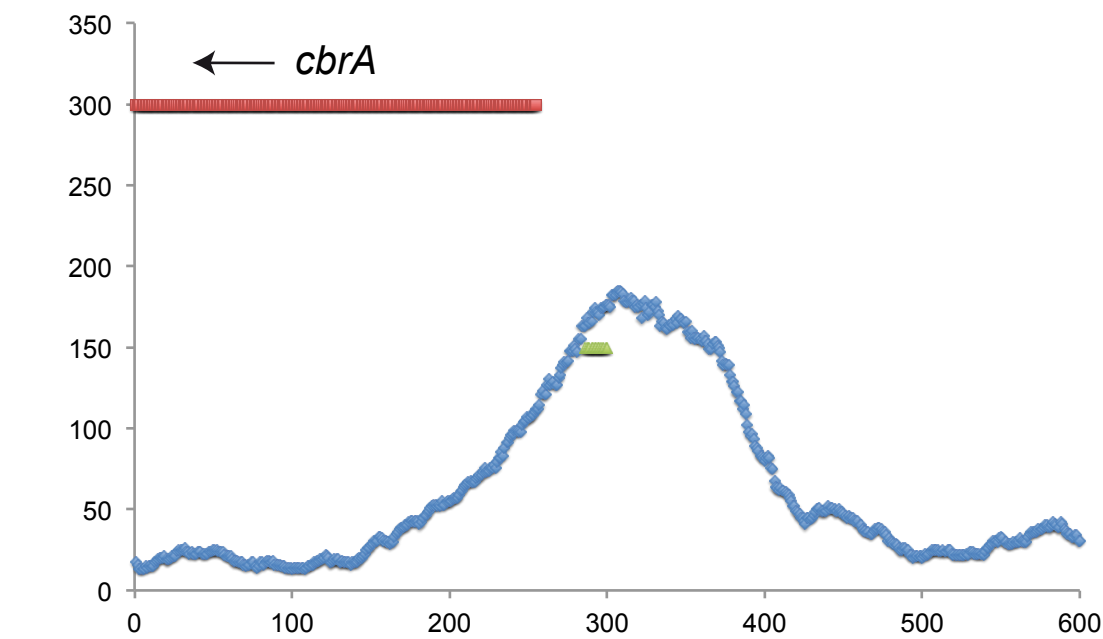

B

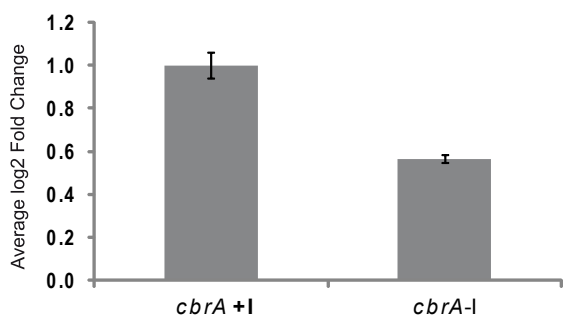

C

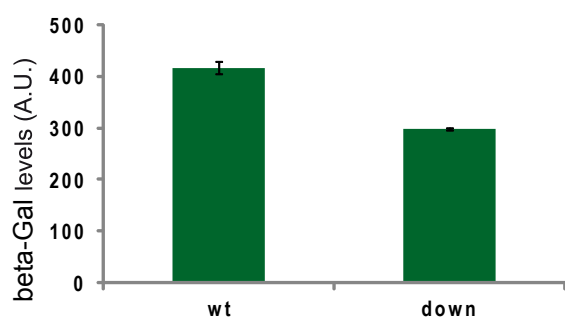

Supplement: S6 Fig — No transcriptional start site has been experimentally identified for cbrA. Green lines represent predicted CtrA binding site. B. Fold change in cbrA expression in cells after depletion of CtrA (-I, IPTG) for two hours relative to control cells expressing CtrA (+I). Expression of cbrA was normalized to the expression of the control gene smc00128. Shown are data from a representative biological replicate. Error bars indicate standard error. C. Beta-galactosidase activity assay using a LacZ fusion of the cbrA promoter in cells after depletion of CtrA (-I, IPTG) for two hours relative to control cells expressing CtrA (+I). (PDF) [file pgen.1005232.s015.pdf]

FIGURE S7

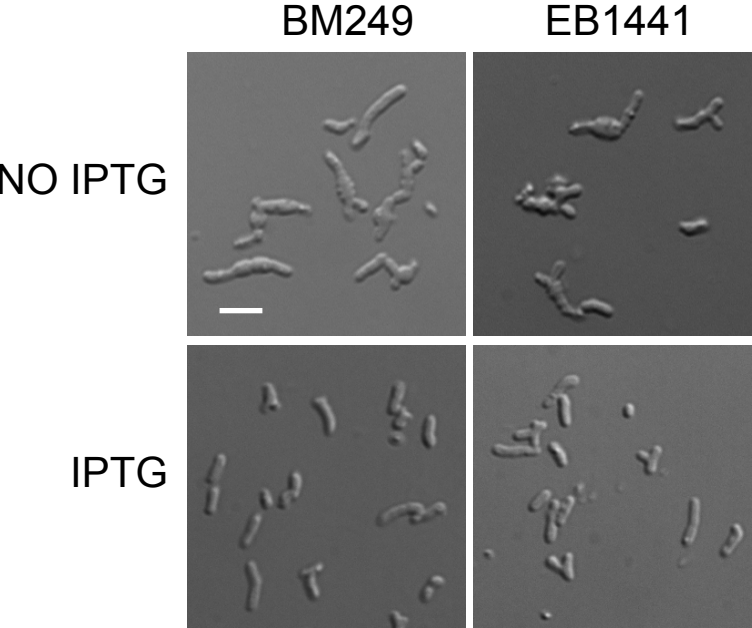

Supplement: S7 Fig — (PDF) [file pgen.1005232.s016.pdf]
